# Progress, Challenges, and Opportunities in Ionic Liquid–Modified Polymer Membranes for CO_2_ Separation

**DOI:** 10.1021/acsomega.5c08808

**Published:** 2026-02-19

**Authors:** Julia A. Piotrowska, Michael Harasek, Katharina Bica-Schröder

**Affiliations:** † TU Wien, Institute of Applied Synthetic Chemistry, Getreidemarkt 9/166, 1060 Vienna, Austria; ‡ TU Wien, Institute of Chemical, Environmental and Bioscience Engineering, Getreidemarkt 9/163, 1060 Vienna, Austria

## Abstract

Driven by the critical challenge of mitigating anthropogenic CO_2_ emissions, the field of carbon capture and utilization (CCU) has undergone rapid development. Among these, membrane-based CO_2_ separation stands out for its energy efficiency and ease of industrial implementation. However, challenges such as the selectivity-permeability trade-off and plasticization limit its effectiveness. Functionalizing membranes with ionic liquids (ILs) has emerged as a promising strategy that takes advantage of the ILs’ high CO_2_ solubility, stability, and tunability to enhance separation performance. Beyond CO_2_ capture, ILs also serve as key promoters in CO_2_ conversion by facilitating various reactions. This Review summarizes recent progress in IL-functionalized polymer membranes, highlighting advances in polymerized ILs, mixed matrix membranes, and hollow fiber configurations. Representative studies are discussed to illustrate how IL incorporation can improve gas transport, selectivity, and stability and enable integrated systems for simultaneous CO_2_ capture and catalytic transformation. Remaining technical challenges, including IL leaching, polymer compatibility, and thermal constraints, are identified, providing guidance for future design and optimization of next-generation IL-membrane platforms for CO_2_ separation and conversion.

## Graphical abstract

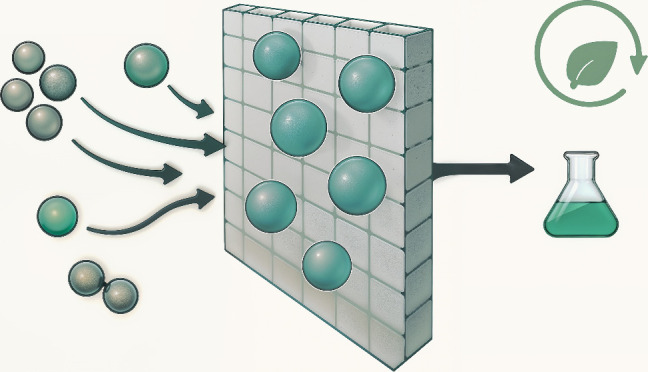

## Introduction

To address the challenges arising from anthropogenic carbon dioxide (CO_2_) emissions, considerable attention has been dedicated to the advancement of carbon capture and utilization (CCU) technologies. The CCU approach integrates both the capture of CO_2_ and its conversion into value-added products.^1–4^

One of the most promising methods for CO_2_ separation and conversion involves the use of membranes, particularly those based on polymers. These membranes have garnered considerable attention due to their ease of fabrication, scalability, and suitability for integration into industrial processes. Moreover, their high tunability and mild operation conditions position them as relevant candidates to replace the conventional CO_2_ separation technologies, such as amine scrubbing or pressure swing adsorption.^5,6^ Membranes offer several advantages over the traditional methods, including lower energy consumption, a more compact design, and reduced waste production—features aligned with Twelve Principles of Green Chemistry, particularly energy efficiency and waste production.^7^ Despite this potential, critical challenges—such as the selectivity-permeability trade-off, limited thermal stability, and membrane plasticization—continue to limit their broader implementation and must be strategically addressed to render them competitive with state-of-art technologies.^8–10^

The integration of ILs, which can be defined as organic salts with melting points below 100 °C, into polymeric membranes has emerged as a promising approach to enhancing the CO_2_ separation. Due to their significantly higher CO_2_ solubility compared to other gases and their tunable molecular structure, ILs are considered highly attractive candidates for improvement of membrane-based gas separation applications.^11,12^ The compelling impact of ILs on the CO_2_ separation has been extensively investigated and corroborated by plentiful researchers. This focus stems from their extraordinary properties, including high solubility of CO_2_, negligible vapor pressure, which reduces the risk of solvent loss, as well as their exceptional thermal and chemical stability. Moreover, their tunable nature allows for the customization of physical and chemical properties, tailored to specific CO_2_ separation applications.^13–15^

In addition, the chemical composition and molecular structure of ILs play decisive roles in determining membrane performance. The choice of cation affects CO_2_ solubility and membrane compatibility through variations in polarity, hydrogen-bonding capability, and steric effects. The anion typically governs the strength and specificity of CO_2_ interactions, with basic or nucleophilic anions (e.g., acetate, amino-functional groups) promoting chemisorption, whereas weakly coordinating anions (e.g., BF_4_^–^, PF_6_^–^) predominantly support physisorption. Side-chain length and functional groups on the cation influence the viscosity, free volume, and IL–polymer miscibility, all of which directly impact the permeability and selectivity. Furthermore, the overall ion-pair structure determines IL mobility within the polymer matrix, the extent of polymer plasticization, and the formation of preferential transport pathways.^14,16,17^

Efficient separation of CO_2_ can be achieved via both physical and chemical interactions provided by ILs, depending on their composition. Physically, their high CO_2_ solubility, caused by favorable interactions between CO_2_ molecules and ionic species of ILs results in enhanced CO_2_ uptake. Chemically, ILs—particularly when functionalized with amino-based groups or acetate ions—can form reversible covalent bonds with CO_2_ and can absorb it via different intermediates.^3,18^

Beyond capturing and separation of CO_2_, ILs can also play a crucial role in its catalytic conversion. They have been recognized as effective CO_2_ transformation promoters, capable of activating CO_2_ molecules, stabilizing reaction intermediates, enhancing proton transfer, and improving reaction selectivity.^19,20^ The high efficiency of ILs in CO_2_ conversion is attributed to their strong affinity for various chemical species, along with their exceptional thermal and chemical stability.^21^ The role of ILs can be versatile, ranging from catalysts and cocatalysts to solvents, offering a tunable reaction medium for chemical, electrochemical, thermal, and biological CO_2_ utilization. Some of the most prominent CO_2_ conversion reactions involving ILs include CO_2_ hydrogenation, which yields products such as methanol, formic acid, or methane,^20,22^ as well as the reduction of CO_2_ to carbon monoxide (CO).^23–26^ Moreover, ILs can also facilitate carboxylation and carbonylation reactions, as well as the CO_2_ cycloaddition to epoxides, leading to formation of cyclic carbonates under mild conditions.^27–29^

Consequently, the integration of ILs with membrane technology has been recognized as a promising strategy toward the simultaneous and efficient CO_2_ separation combined with its conversion, as illustrated in Figure 1

**Figure 1:**
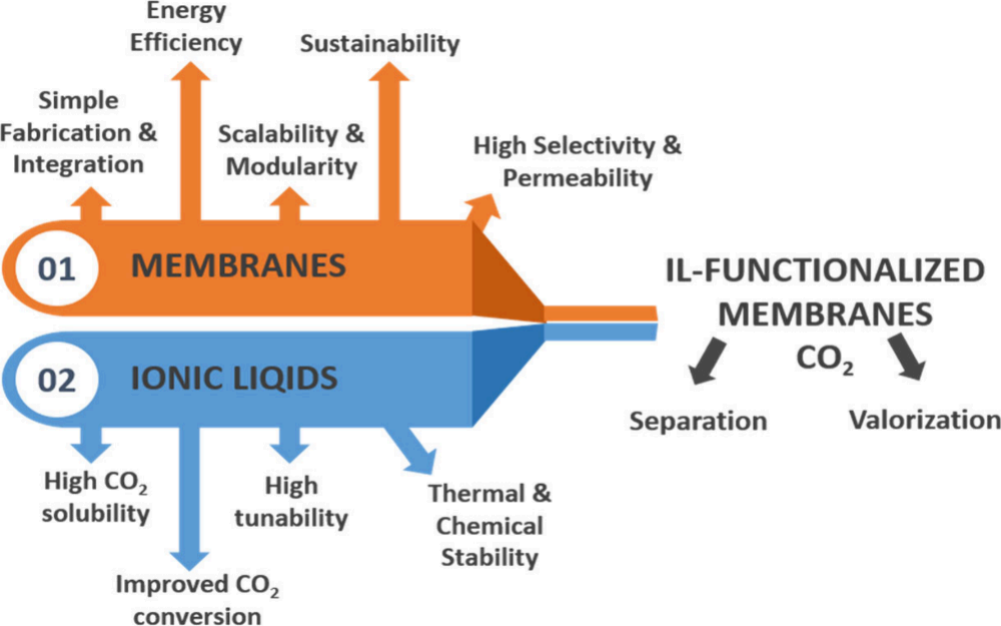
Graphical representation of ILs and membrane technology: advantages and combination.

However, despite their numerous advantages, the high viscosity and costly production of ILs continue to hinder their widespread application in membrane-based CO_2_ separation. Common synthesis routes, such as quaternization of organic cations followed by anion exchange or metathesis reactions, often require multiple steps, high-purity reagents, and significant energy input, while generating chemical waste, all of which contribute to higher costs and raise sustainability concerns.^30^ Estimates of IL production costs vary depending on scale and purity, but reported figures are generally in the range of 100 USD – 1,000 USD per kilogram for specialty ILs, significantly higher than conventional amines used in carbon capture (e.g., monoethanolamine).^31,32^ Additionally, the environmental footprint of IL production, including the energy input and potential toxicity of precursors or waste streams, must be considered. Nevertheless, IL-functionalized membranes offer operational benefits, including reduced solvent loss, lower corrosion risk, and the potential for integration with catalytic conversion, which may partially offset these limitations over long-term operation. To overcome these challenges, IL-functionalized membranes are being further optimized through the incorporation of additional compounds, aiming to enhance both performance and cost-effectiveness.^11,33,34^ Recent advancements in CO_2_ separation have led to the development of novel composite polymeric membranes functionalized with ILs, resulting in thin film composite (TFC) structures. Figure 2 illustrates key emerging technologies in this field, including four promising approaches aimed at enhancing the performance of IL-functionalized membranes, which will be discussed in detail in this Review.

**Figure 2:**
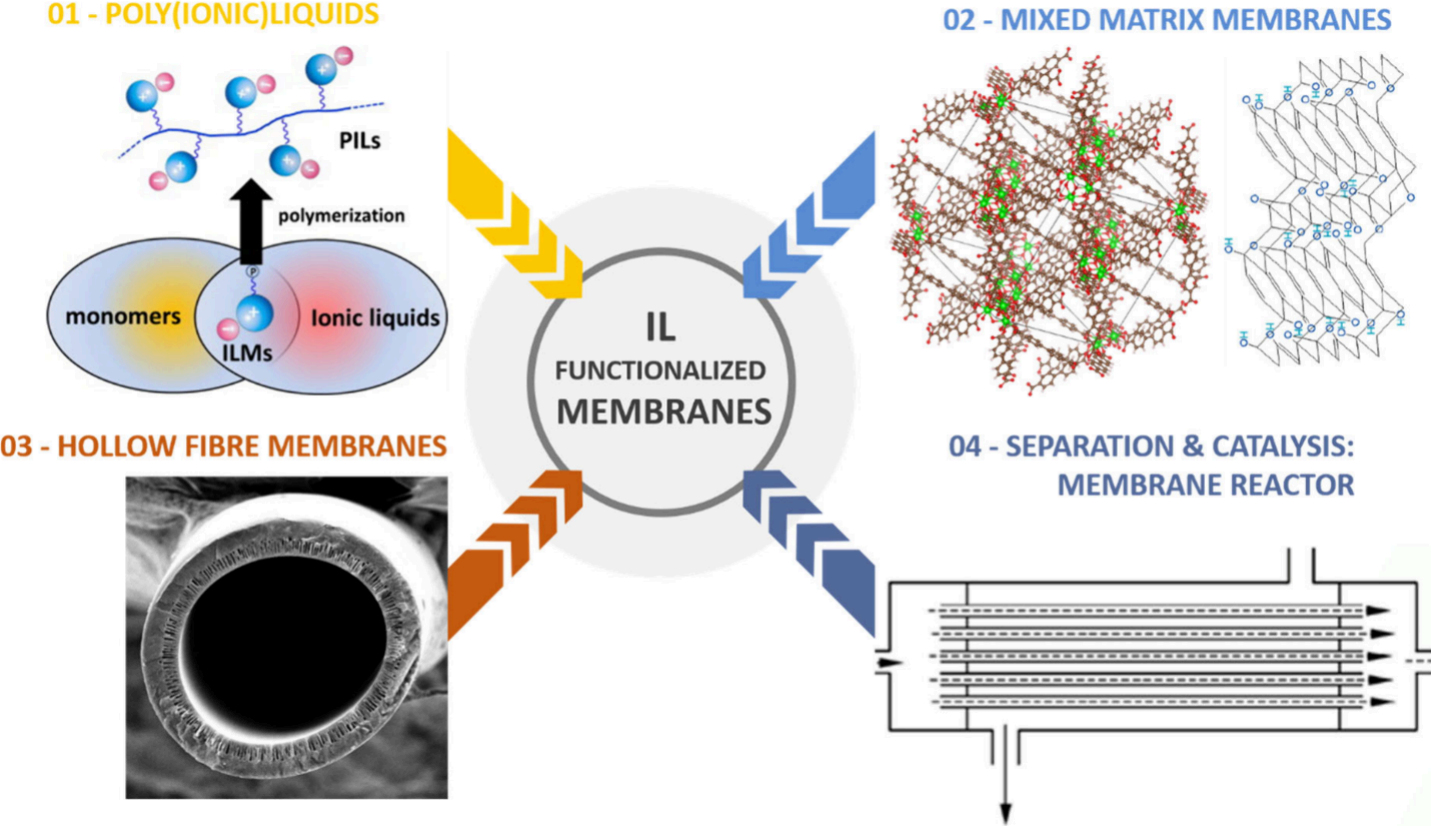
Investigated strategies to improve IL-functionalized membrane gas separation performance. Adapted from ref 142 with permission from Chem. Mater. Copyright (2006) American Chemical Society, Also adapted from ref 143 with permission from Chem. Mater. Polzm. Sci. Copyright (2013) Elsevier.

- First, membranes can be enriched with chemically functionalized ILs, leading to the formation of poly­(ionic liquid)-based membranes (PILs).^35,36^

- Another broad and widely studied strategy involves the formation of mixed matrix membranes (MMMs)^37–39^ which incorporate ILs or PILs, along with additional functional materials, such as graphene oxide (GO),^40–42^ metal–organic frameworks (MOFs)^43,44^ and covalent organic frameworks (COFs) or zeolitic imidazolate frameworks (ZIFs),^45^ known for their exceptional properties.
- In addition to these material-oriented approaches, researchers have also explored alternative membrane configurations. It is important to note that this category differs from the previous two, as it refers to membrane architecture rather than IL molecular design. Among these, hollow fiber membranes (HFMs) have received particular attention due to their high surface-area-to-volume ratio and the resulting improvements in gas separation efficiency.^14,46^

- Finally, combining CO_2_ separation with simultaneous CO_2_ conversion using IL-based catalytic membranes represents an emerging and highly synergistic approach. This integration of IL-based catalysis with membrane separation has the potential to significantly contribute to carbon capture and utilization (CCU). These trends underscore the rapid progress in the field and highlight the strong potential of IL-functionalized membranes for efficient and multifunctional CO_2_ separation technologies.^47^

This review summarizes recent progress in carbon capture and utilization (CCU) technologies using ionic liquid (IL)–functionalized polymer-supported membranes. The integration of ILs into membrane structures has been widely explored to exploit their tunable chemical properties, high affinity for carbon dioxide, and potential catalytic activity for enhancing separation and conversion processes. Membrane-based approaches offer advantages over conventional CO_2_ capture or separation methods, including modularity, lower energy requirements, and opportunities for process integration.^48^ Recent studies on IL-modified membranes are discussed with emphasis on materials design strategies and their role in enabling both CO_2_ separation and catalytic utilization. Key limitations, including the permeability–selectivity trade-off and operational stability, are critically assessed alongside further considerations such as IL synthesis complexity, material costs, and environmental impacts. By consolidating advances and challenges, this review provides a comprehensive overview of the state-of-the-art and identifies research directions needed to advance IL-functionalized membranes toward practical CCU applications.

## Poly(ionic liquid)-Based Membranes

Poly­(ionic liquids) (PILs) are a specialized class of polyelectrolytes in which each repeating unit contains an ionic liquid moiety, either integrated into the polymer backbone or attached as a pendant side group. Synthesized through the polymerization of IL monomers bearing a polymerizable group, PILs can be considered as the macromolecular analogues of ILs. They form distinct charged polymeric structures that combine the unique physicochemical properties of ILs with the mechanical strength, film-forming ability, and processability of traditional polymers.^49–51^

Notably, when compared to their monomeric counterparts, PILs usually exhibit significantly higher CO_2_ uptake,^50^ along with enhanced mechanical stability, processability and tunability of their meso- and nanostructures. One major disadvantage of ILs in membranes is their tendency to leach out, especially under humid or high-pressure conditions. PILs immobilize the ionic species within the polymer backbone, thus preventing leaching and ensuring more consistent separation performance.^13,52,53^ Recent advancements in PIL-based membranes for CO_2_ separation have significantly enhanced their performance in CO_2_ separation. Current research focuses on the development of PIL-IL composites, the incorporation of functionalized nanomaterials, and molecular engineering of co-PILS.^13,49,50,54,55^ The combination of ILs with membranes can be achieved through various approaches and configurations,^51^ but to maintain clarity, this chapter primarily focuses on composites in which PILs are cast onto polymeric supports, unless otherwise specified. Below, several representative examples are presented to illustrate the growing interest in PIL-functionalized membranes.

Development and functionality of composite membranes containing PILs based on imidazolium and pyridinium polycations paired with various anions, such as hexafluorophosphate, tetrafluoroborate, and bis­(trifluoromethylsulfonyl)­imide was recently studied by Otvagina et al.^56^ The synthesized PILs were cast onto a microfiltration fluoroplastic membrane to form composite polymer membranes. Compared to neat ILs, PILs showed superior CO_2_ physisorption. The type of anion influenced IL-functionalized composite membrane performance significantly, with bis­(trifluoromethylsulfonyl)­imide ([NTf_2_]^−^) anions yielding (along with 1-vinyl-3-butylimidazolium—[C_4_vim]—cation) the optimal CO_2_/N_2_ separation selectivity (19.1) and CO_2_ permeance (17 Barrer), as given in Table 1. Future work should assess the influence of water on the membrane performance and investigate polycations with carboxylate anions for improved CO_2_ absorption.

**Table 1:** Comparison of Gas Permeation (Gas Permeances (P) and Selectivities (α)): Membranes Functionalized with PILs^a^

No.	Membrane	S/M	P_feed_ (bar)	PCO_2_ (GPU)	PN_2_ (GPU)	PCH_4_ (GPU)	αCO_2_/N_2_(−)	αCO_2_/CH_4_(−)	ref
1	poly([C_4_vim][NTf_2_])	S	1.3	17*	0.9*	0.9*	19.1	18.9	^56^
	poly([C_4_vim][Br])			15*	1*	0.7*	15.3	21.4	
									
2	poly([VCmim][Br])	M	2	7	0.1		72		^57^
	poly([VCNmim][Br])			29	0.4		72		
									
3	Pure PSF	M	2	21	0.6**		33		^58^
	poly([VCNmim-*co*-AAm][Br])/PSF; IL:AM 1:1			52	0.8**		66		
	poly([VCNmim-*co*-AAm][Br])/PSF; IL:AM 1:3			76	1.4**		53		
	poly([VCmim-COOH-*co*-AAm][Br]/PSF; IL:AM 1:1			43	0.7**		64		
									
4	poly([C_2_amvtaz][Br]-*co*-PAm)/PZEA-Sar; PIL: 10 wt %	M	2	16	0.1		173.3		^59^

a The chemical structures of the ionic liquids summarized in this table are given in the Supporting Information. Gas permeabilities are expressed in GPU; for reference, 1 GPU corresponds to 3.35 × 10^–10^ mol m^–1^ s^–1^ Pa^–1^, which is equivalent to 1 Barrer = 10^–10^ cm^3^(STP) cm cm^–2^ s^–1^ cmHg^–1^. Definitions: S/M, single or mixed gas conditions; P_feed_, driving pressure difference; *, gas permeability given in Barrer; **, data estimated for the comparison, not given in the article; poly­([C_4_vim]­[NTf_2_]), poly­(1-vinyl-3-butylimidazolium bis­(trifluoromethylsulfonyl)­imide); poly­([C_4_vim]­[Br]), poly­(1-vinyl-3-butylimidazolium bromide); poly­([VCmim]­[Br]), poly­(1-carboxymethyl-3-vinylimidazolium bromide); poly­([VCNmim]­[Br]), poly­(1-cyanomethyl-3-vinylimidazolium bromide); PSF, polysulfone; poly­([VCNmim-*co*-AAm]­[Br], poly­(1-cyanomethyl-3-vinylimidazolium bromide-*co*-acrylamide); poly­([VCmim-COOH-*co*-AAm]­[Br]), poly­(1-carboxymethyl-3-vinylimidazolium bromide-*co*-acrylamide); poly­([C_2_amvtaz]­[Br]-*co*-PAm), poly­(4- aminoethyl-1-vinyl-1,2,4-triazolium) bromide-*co*-polyacrylamide; PZEA-Sar, 2-(1-piperazinyl) ethylamine sarcosine.

In 2023 Zhang et al.^57^ reported the successful synthesis of vinyl imidazolium-based PILs, containing cyano (−CN) and carboxy (−COOH)-based side chain groups. PILs-functionalized membranes were obtained by casting solutions of PILs blended with polyacrylamide (PAM) over a polysulfone (PSF) support to form thin film composites (TFCs). Composite membranes exhibited enhanced hydrophilicity, especially for cyano-based PILs. Both PILs achieved peak CO_2_ selectivity at 10 wt % polymer concentration, with cyano-based PIL showing higher CO_2_ permeance than carboxyl-based one. This difference can be explained by the distinct molecular interactions of the side chains: the – CN group, being strongly dipolar, enhances CO_2_ solubility while maintaining microfree volume in the polymer matrix, facilitating faster gas diffusion. In contrast, −COOH groups engage in extensive hydrogen bonding, increasing polymer density and reducing free volume, which limits gas transport. As a result, cyano-functionalized PILs combine higher CO_2_ permeance with good selectivity. As shown in Table 1, the best-performing membrane (modified with poly­(1-cyanomethyl-3-vinylimidazolium bromide) – poly­([VCNMim]­[Br]) reached CO_2_ permeance of 29 GPU and CO_2_/N_2_ selectivity of 72, demonstrating competitiveness with state-of-the-art PIL-based membranes (see Figure 3). Despite the superior selectivity of membranes synthesized by Zhang, further tuning of PIL side chains is necessary to surpass Robeson’s upper bound (2008).

**Figure 3:**
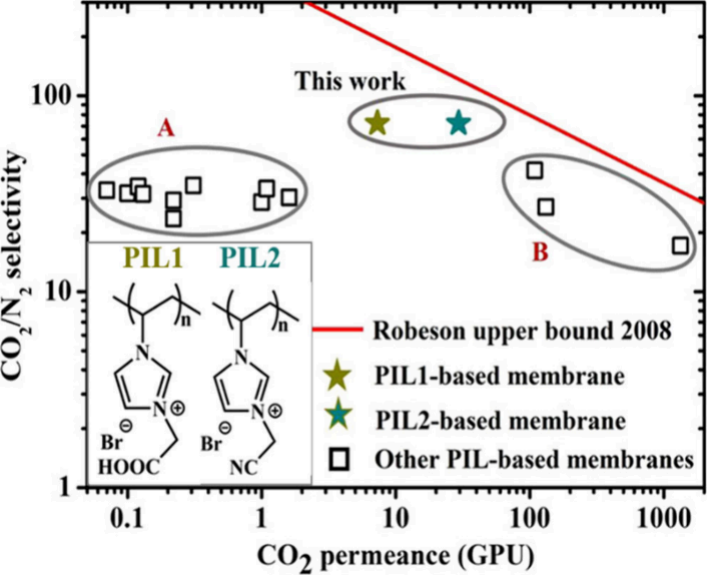
Comparison of the CO_2_/N_2_ separation performance of PIL-based membranes, visualized on a Robeson plot (2008). Reproduced from ref 57 with permission from Ind. Eng. Chem. Res. Copyright (2023) American Chemical Society.

A further example of ILs polymerization, carried out by Zhang’s group,^58^ focused on the development of PILs via copolymerization of imidazolium-based monomers containing two distinct functional groups: acrylamide (AM) and butyl acrylate (BA). The best-performing TFCs, developed using a PSF support and synthesized co-PILs containing cyanomethyl-3-vinylimidazolium bromide and AM in a 1:3 ratio, exhibited a CO_2_ permeance of 76 GPU and a CO_2_/N_2_ selectivity of 53, surpassing neat PSF supports by 262% and 61%, respectively (see Table 1). Notably, AM-based monomers delivered the strongest enhancement, attributed to their hydrophilicity and the presence of reactive hydrogen (H_2_), which facilitated more effective CO_2_ transport.

Finally, Zhang et al. investigated also the impact of incorporating the mobile carrier, 2-(1-piperazinyl) ethylamine sarcosine (PZEA-Sar), as illustrated in Scheme 1, and presented the free radical synthesis of two novel amino functionalized PILs.^59^ They were used to develop highly hydrophilic membrane composites by casting of PZEA Sar PILs based solution over a PSF support.

**Scheme 1:**
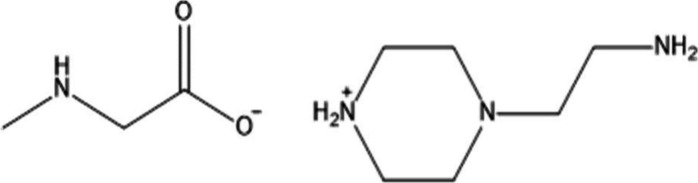
Molecular Structure of PZEA-Sar

These PILs were used to develop highly hydrophilic membrane composites by casting PIL solutions over poly­(sulfone) (PSF) supports. The choice of amino-functionalized PILs was supported by prior studies, such as Sun and co-workers’ work on poly­[1-(p-vinylbenzyl)-3-methylimidazolium glycinate] (P­([VBMI]­[Gly])), which confirmed the usefulness of amino-functionalized PILs for CO_2_ separation and could be processed into porous membranes via supercritical foaming.^60^ Moreover, the usefulness of amino-functionalized ILs has been further demonstrated by Yin et al.,^61^ who investigated amino-functionalized ionic-liquid-grafted covalent organic frameworks for high-efficiency CO_2_ capture and conversion. The composites synthesized by Zhang exhibited efficient CO_2_/N_2_ separation and the incorporation of PZEA-Sar further enhanced their performance, with the best-performing membrane achieving a CO_2_/N_2_ selectivity of 173.5 (see Table 1). This significant enhancement can be attributed to the amino groups of PZEA acting as reversible CO_2_ carriers, facilitating facilitated transport through transient carbamate formation and improving CO_2_ solubility without compromising membrane permeability. This highlighted the potential of amino-functionalized carriers such as PZEA-Sar to enhance membrane performance and support sustainable design.

## Mixed Matrix Membranes

One of the intensively studied strategies to improve the performance of IL-functionalized TFCs is the development of mixed matrix membranes (MMMs). These advanced hybrid materials integrate a polymer matrix with ILs or PILs and a variety of fillers, including MOFs and their subclass, ZIFs, as well as COFs, zeolites, and GO-based materials. Selected inorganic salts such as alkali metal (e.g., LiBF_4_, lithium bis­(trifluoromethanesulfonyl)­imide Li­[NTf_2_], K_2_CO_3_ or transition metal (Cu^2+^ and Zn^2+^) salts are often incorporated, as illustrated in Figure 4. These additives are introduced to enhance key performance parameters for the CO_2_ membrane-based separation, including gas selectivity, permeability, mechanical strength, and interfacial compatibility between the polymer matrix and dispersed phase. Furthermore, they can impart specific functionalities, such as facilitated CO_2_ transport, by improving chemical affinity or introducing additional diffusion pathways. However, it is noteworthy that salts like Li­[NTf_2_], despite their benefits, raise concerns due to persistence and toxicity.^62^ Overall, the incorporation of additives addresses the limitations of pristine polymers or fillers, thereby enabling more efficient and robust membrane-based CO_2_ separation.^63–65^

**Figure 4:**
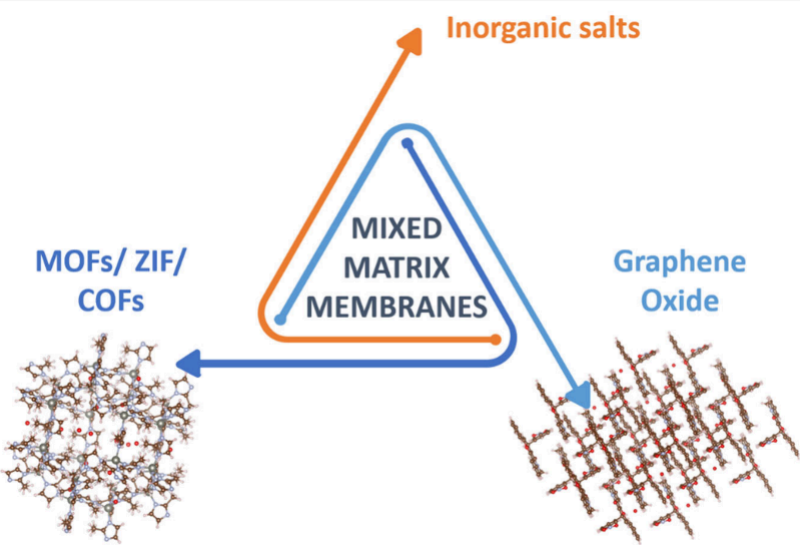
Illustrated examples of possible MMM additives, including MOFs, ZIFs, COFs, GO, and selected inorganic salts, along with their representative molecular structures.

### Inorganic Additives

Nikolaeva et al.^66^ proposed a novel approach for modifying PIL-based matrices by developing MMMs incorporating salts, forming PIL/IL/salt composites. They examined the performance of PIL-based composites synthesized using mixtures of zinc salts – Zn­[NTf_2_]_2_, PILs – poly­(diallyldimethylammonium) bis­(trifluoromethylsulfonyl)­imide (poly­[DADMA]­[NTF_2_]), and blended ILs: N-butyl-*N*-methyl pyrrolidinium bis­(trifluoromethylsulfonyl)­imide ([Pyrr_14_]­[NTf_2_]) with Zn­(NTF_2_)_2_, which formed an active, self-standing membrane or were coated onto polyimide (PI)-based nanofiltration membranes to create TFCs. The addition of ILs enhanced the CO_2_ diffusivity, while the inclusion of Zn^2+^ increased the equilibrium solubility selectivity by 50% compared to the neat PIL, attributed to disruption of polymer chain packing. In composites containing all three components, PIL, IL, and Zn^2+^ – CO_2_ sorption was further improved through synergistic interactions with [NTf_2_]^−^ anions and Zn^2+^ cations, achieving a maximum equilibrium CO_2_ sorption capacity of 2.77 cm^3^(STP) cm^–3^ bar^–1^ (for a PIL:IL:Zn^2+^ ratio of 9:6:9). As shown in Table 2, the best CO_2_ separation performance was observed for membranes containing 6 wt % [Pyrr_14_]­[NTf_2_] without Zn­(NTF_2_)_2_, reaching a CO_2_ permeance of 166 Barrer and a CO_2_/N_2_ selectivity of 30.5. The performance enhancement observed for membranes containing 6 wt % [Pyrr_14_]­[NTf_2_] can be attributed to the role of the ionic liquid as a molecular mobility enhancer within the PIL matrix. [Pyrr_14_]­[NTf_2_] is a low-viscosity, highly CO_2_-philic ionic liquid; when blended into the PIL, it increases segmental mobility and free volume by partially plasticizing the polymer chains. This reduces the energy barrier for CO_2_ diffusion, thereby increasing the permeance. At the same time, the presence of the highly fluorinated [NTf_2_]^−^ anion increases the CO_2_ solubility through favorable Lewis acid–base and quadrupole–anion interactions. In the absence of Zn­(NTf_2_)_2_, which mainly contributes to solubility selectivity via coordination with the PIL backbone, the permeance increase is therefore dominated by the IL-induced enhancement of diffusivity and CO_2_ affinity. Additionally, mixed-gas separation under humid conditions showed a 10% performance improvement. The enhanced performance of PIL/IL/Zn^2+^-based TFC membranes under process-relevant conditions simulating real flue gas environments highlights their potential for practical CO_2_ capture.

**Table 2:** Comparison of Gas Permeation Results (Gas Permeances (P) and Selectivities (α)): Membranes Functionalized with Mixed Matrix Membranes^a^

No.	Membrane Description	P_feed_ (bar)	T (°C)	S/M	PCO_2_ (GPU)	PN_2_ (GPU)	PCH_4_ (GPU)	αCO_2_/N_2_(−)	αCO_2_/CH_4_ (−)	ref
Inorganic Additives										
1	poly([DADMA][NTf_2_]):[Pyrr_14_][NTf_2_]:Zn[NTf_2_]_2_ 9:0:0	1	25	M, 15% CO_2_, 85% N_2_	7.6*	0.4*	0.3*	17.6	25.3	^66^
	poly*([DADMA][NTf_2_]):[Pyrr_14_][NTf_2_]:Zn[NTf_2_]_2_ 9:6:0				166*	5.4*	10.1*	30.5	16.4	
	poly([DADMA][NTf_2_]):[Pyrr_14_][NTf_2_]:Zn[NTf_2_]_2_ 9:6:1				80.9*	7.2*	18.9*	11.2	4.3	
										
2	PIL/[C_2_mim][NTf_2_]/SAPO-34; PIL polymerization deg.: 87; IL: 16 wt %; SAPO-34: 20 wt %	1	21	S	47*		1.1*		42	^69^
Metal Organic Frameworks										
3	PSF/[C_3_PTMSam][Ac], IL: 30 wt %	10	25	S	16.0*	0.4*	0.4*	39.0	39.0	^76^
	PSF/[C_3_PTMSam][Ac]/ ZIF-67; IL: 30 wt %; ZIF-67: 30 wt %				22.3*	0.3*	0.3*	74.5	72.1	
										
4	Pure PAP	1	25	S	1056	49	110	22	10	^77^
	PAP/[C_2_mim][NTf_2_]				1106	41	95	27	12	
	PAP/ [C_2_mim][NTf_2_]/ZIF-8; ZIF-8: 10 wt %				1017	31	81	33	13	
										
5	[P_66614_][Cl]/Cu_*x*_Mg_*x*_ MOF; MOF: 0.2 wt %; IL: 0.8 wt %	2	25	S	2937*	88.2*		33.3		^78^
										
6	PI/[C_3_mim][Br]/MIL-101(Cr); MOF: 10 wt %	0.7	30	S	11.8*	0.8* **		15.2		^79^
	PI/[C_3_mim][Br]/MIL-101(Cr); MOF: 20 wt %				9.9 *	0.4* **		26.9		
	PI/[C_3_mim][Br]/MIL-101(Cr); MOF: 30 wt %				7.9 *	0.4* **		19.7		
										
7	Pure Pebax 1657	10	25	S	98.3*	1.9*	3.8*	48.2	21.6	^80^
	Pebax/[APTMS][Ac]/UiO-66; UiO-66: 10 wt %				115.4*	2.1*	4.2*	52.6	23.2	
	Pebax/[APTMS][Ac]/UiO-66; UiO-66: 20 wt %				143*	2.3*	4.5*	55.0	25.4	
	Pebax/[APTMS][Ac]/UiO-66; UiO-66: 30 wt %				98.3*	1.9*	5.0*	61.1	28.3	
Graphene Oxide										
8	[C_2_mim][Ac]	1	25	S				116	33	^85^
	[C_2_mim][Ac]/GO; GO: 0.5 wt %							130	39	
										
9	Pure Pebax	4	25	S	722.6	23.7		30.5		^86^
	0.05 wt % GO-[APmim][Br]**-**NH_2_/Pebax TFC				905.4	20.2		44.8		
	0.2 wt % GO-[APmim][Br]**-**NH_2_/Pebax TFC				783.3	18.2		43.0		
										
10	poly([DADMA][2-CNpyr])/GO, Rel. Humid.: 40%	1	22	S	3092	2.6		1189		^87^
11	Pure PSF	3	30	S	12	0.3	0.4	0.4	47	^88^
	PSF/C3IL/N-GO				130	2.7	–3.7	3.6	–36	
	PSF/C6IL/N-GO				100	1.8	2.7	2.7	–37	
										
12	GO/[C_1_mim][BF_4_]	0.5	25	S	13.85					^89^
										
13	GO/CNT/[BMIM][BF4]; IL- 20 mg/m^2^; humidified conditions	0.2	80	M	600	9.7**		62		^90^

a Gas permeabilities are expressed in GPU; for reference, 1 GPU corresponds to 3.35 × 10^–10^ mol m^–1^ s^–1^ Pa^–1^, which is equivalent to 1 Barrer = 10^–10^ cm^3^(STP) cm cm^–2^ s^–1^ cmHg^–1^. Definitions:*. gas permeability given in Barrer; **, data estimated for the comparison, not given in the article; P_feed_, driving pressure difference; T, temperature; S/M, single or mixed gas conditions; poly­([DADMA]­[NTf_2_]), poly­(diallyldimethylammonium) bis­(trifluoromethylsulfonyl)­imide; [Pyrr_14_], N-butyl-*N*-methyl pyrrolidinium; [C_2_mim]­[NTf_2_], 1-ethyl-3 methylimidazolium bis­(trifluoromethylsulfonyl)­imide; SAPO-34, silicoaluminophosphate-34; PSF, polysulfone; [C_3_PTMSam]­[Ac], 3-(trimethoxysilyl) propan-1-aminium acetate; PAP, poly­(1-allyl-3-methylimidazoliumbis­(trifluoromethanesulfonyl)­imide-*co*-poly­(ethylene glycol) methyl ether methacrylate; [P_66614_]­[Cl], trihexyltetradecylphosphonium chloride; PI, polyimide; [C_3_mim]­[Br], 1-propyl-3-methyl-imidazolium bromide; [APTMS]­[Ac], 3-(trimethoxysilyl)­propan-1-aminium acetate; [C_2_mim]­[Ac], 1-ethyl-3-methylimidazolium acetate; GO, graphene oxide; [APmim]­[Br]**-**NH_2_, 1-(3-aminopropyl)-3-methylimidazolium bromide; poly­([DADMA]­[2-CNpyr]), poly diallyldimethylammonium 2-cyanopyrrolide; C3IL, trimethylene linker; C6IL, hexamethylene linker; [C_1_mim]­[BF_4_], 1-butyl-3-methylidazolium tetrafluoroborate; [BMIM]­[BF_4_], 1-butyl-3-methylimidazolium tetrafluoroborate.

As a potential third inorganic component, zeolites can also be considered attractive, low-cost, highly abundant, and environmentally benign additives for PIL/IL-based MMMs. They can be defined as crystalline, microporous aluminosilicate materials with a well-defined, three-dimensional framework structure, widely used as molecular sieves, ion exchangers, and catalysts due to their high surface area, thermal stability, and selective adsorption properties.^67,68^

Dunn et al.^69^ demonstrated this by synthesizing a membrane using curable IL prepolymers (from poly­(4-chloromethylstyrene) (PCMS)), the imidazolium-based IL—1-ethyl-3-methylimidazolium bis­(trifluoromethylsulfonyl)­imide ([C_2_mim]­[NTf_2_]) and silicoaluminophosphate-34 (SAPO-34) zeolite, over renewable regenerated cellulose-based support. The use of IL prepolymers gave control over the casting solution’s penetration, gelation time, and CO_2_/CH_4_ separation efficiency. The chain length was modified by varying the oligomeric PCMS precursors synthesized via reversible addition–fragmentation chain-transfer (RAFT) polymerization. These prepolymers, which possess cross-linking functionalities, showed reduced support penetration compared to that of conventional IL monomers. The degree of polymerization (x) was crucial, with minimal support penetration achieved at x = 87. In this configuration, with 16 wt % IL and 20 wt % SAPO-34 zeolite, the membrane achieved an optimal CO_2_/CH_4_ selectivity of (42 ± 5) and CO_2_ permeability of (47 ± 1) Barrer (see Table 2). Compared to previous-generation composites,^70^ with small-molecule curable IL monomers, the new membranes demonstrated similar CO_2_/CH_4_ separation, but with improved resistance to support penetration, faster gelation, and reduced susceptibility to additive volatility. The use of SAPO-34 zeolite as a benign and abundant filler further contributed to the material’s practical promise.

### Metal Organic Frameworks

Another wide class of recently explored MMMs components includes metal–organic frameworks (MOFs) and zeolitic imidazolate frameworks (ZIFs)—a MOF subclass with imidazole linkers, both known as coordination polymers. These materials, extensively studied for over 30 years, are crystalline, highly porous frameworks composed of metal ions or clusters and organic ligands, offering exceptional tunability through the combination of diverse nodes and linkers.^41,71,72^ MOFs enhance CO_2_ membrane separation due to their high porosity and adjustable pore sizes, which allow for optimizing interactions with CO_2_ molecules while maintaining high permeability.^73–75^

An interesting study on MMM composite containing ZIFs and ILs was proposed by Yasmeen et al.,^76^ where the room temperature IL—3-(trimethoxysilyl) propan-1-ammonium acetate ([C_3_PTMSam]­[Ac])—was combined with an inorganic filler, ZIF-67. Unlike in previously cited studies, PSF functioned as the membrane matrix rather than a support material, forming a freestanding membrane instead of a TFC. Figure 5 illustrates this configuration along with the IL structure, SEM images of the synthesized ZIFs, and molecular structure of ZIF-67. The incorporation of ZIF into PSF-IL membranes significantly improved gas separation. Compared to neat PSF-IL with 30 wt % loading, the ZIF-enriched PSF-IL composite showed CO_2_/CH_4_ and CO_2_/N_2_ selectivity increased by 84% and 90%, reaching 72.06 and 74.47, respectively (see Table 2). Well-dispersed fillers enhanced the membrane’s performance, with a strong IL-ZIF synergy: [C_3_PTMSam]­[Ac] offered high CO_2_ affinity, while ZIF-67 provided a large surface area and controlled nanoporosity.

**Figure 5:**
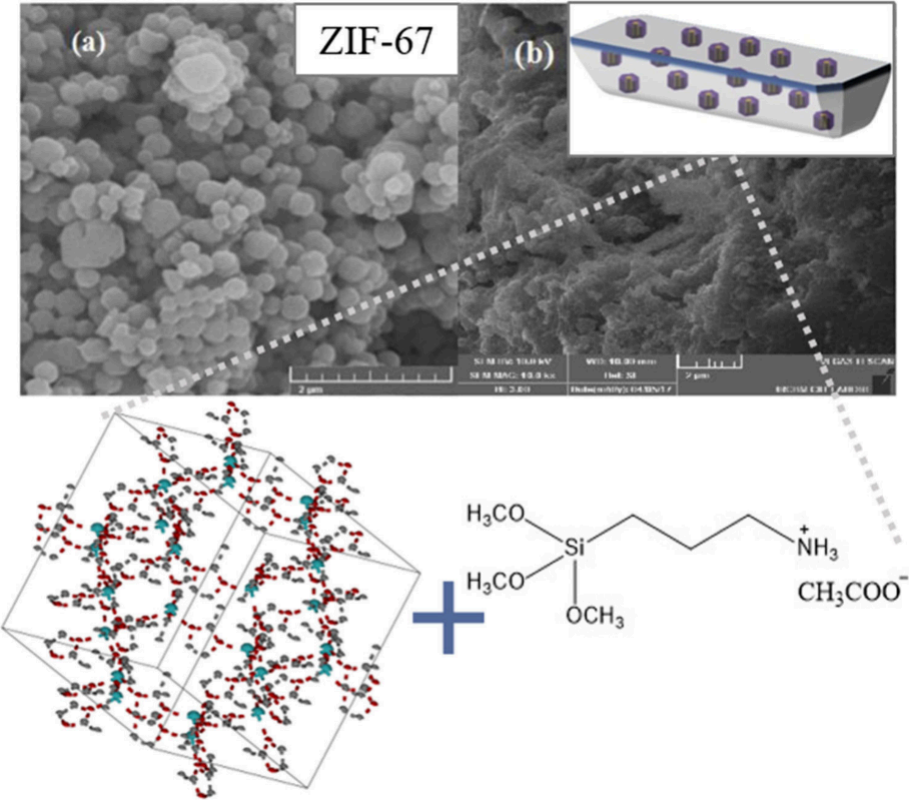
SEM images of (a) ZIF-67 and (b) ZIF-67 modified with RTIL, shown alongside the molecular structures of ZIF-67 and the ionic liquid [C_3_PTMSam]­[Ac]. Adapted from ref 76 with permission from Chem. Eng. Res. Des. Copyright (2020) Elsevier.

Another example of an MMM composite containing ZIFs and ILs was proposed by Kang et al.^77^ In 2024 they presented the design of a thin-film ternary MMM composite, incorporating an IL ([C_2_mim]­[NTf_2_]) and ZIF-8 into a novel comb PIL copolymer, poly­(1-allyl-3-methylimidazoliumbis­(trifluoromethanesulfonyl)­imide-*co*-poly­(ethylene glycol) methyl ether methacrylate (PAP), immobilized on a PSF membrane. PAP was synthesized via free radical polymerization, combining ionic and nonionic CO_2_-philic monomers. The IL enhanced the CO_2_ solubility, while 60 nm-sized ZIF-8 nanoparticles (NPs) served as a size-sieving agent. The polymer structure ensured excellent miscibility and strong chemical interactions without infiltration of PAP/IL into the support, thereby preserving the ZIF-8 crystal structure. The intrinsic pore size of ZIF-8 (3.4 Å) further facilitated the CO_2_ size-selective permeation. The PAP/IL/ZIF composite exhibited excellent CO_2_ separation performance, with the IL enhancing both CO_2_ selectivity and permeance. As shown in Table 2, PAP functionalized only with IL displayed a CO_2_ permeability of 1106 GPU and CO_2_/N_2_ and CO_2_/CH_4_ selectivities of 27 and 12, respectively. Incorporation of 10 wt % ZIF-8 resulted in a slightly reduced CO_2_ permeability of 1017 GPU but improved CO_2_/N_2_ and CO_2_/CH_4_ selectivities of 33 and 13, respectively. Overall, this example highlights how the combined use of ILs and ZIF-8 fillers can achieve a favorable balance between the permeability and selectivity in mixed-matrix membranes.

As an alternative porous filler material, bimetallic, copper and magnesium-based MOF (Cu_*x*_Mg_*x*_) was investigated by Ali et al.^78^ A bimetallic MOF was blended with 0.8 wt % trihexyltetradecylphosphonium chloride ([P_66614_]­[Cl]) to create a MOF/IL composite, supported on a polytetrafluoroethylene (PTFE) porous substrate. The modified membranes were evaluated for CO_2_/N_2_ gas separation, with those containing 0.2 wt % MOF and 0.8 wt % IL achieving the highest CO_2_ permeability (2937 Barrer) and ideal CO_2_/N_2_ selectivity (33.3). The effective combination of the Cu_*x*_Mg_*x*_ MOF and IL significantly enhanced gas separation performance and demonstrated stability even under humid conditions, making it a promising strategy for optimizing membrane-based gas separation.

Various techniques for IL immobilization in MOFs were investigated by Ferreira et al.,^79^ who incorporated two ILs, 1-propyl-3-methyl-imidazolium bromide ([C_3_mim]­[Br]) and 1-butyl-3-methylimidazolium bromide ([C_4_mim]­[Br]) into the MOF structures. The [C_3_mim]­[Br] was added via direct contact (simple addition of MIL to IL solution), while the bulkier [C_4_mim]­[Br] was incorporated using the “ship-in-a-bottle” technique. It is also noteworthy that unlike most of the aforementioned studies, this research did not investigate MMMs supported on porous polymeric materials but instead focused on MMMs used as fillers in a polymeric PI matrix. The applied chromium­(III) terephthalate MOFs, MIL-101­(Cr) and MIL-100­(Cr) both feature zeotypic mesoporous (29 to 34 Å size) cages. The inclusion of ILs improved the mechanical properties of the MMMs. However, the IL/MOF composite had lower CO_2_ adsorption capacity and selectivity compared to pure MOF, likely due to its lower surface area, which was not compensated by the IL’s gas solubilization effect. The MOF loading had a significant impact on performance: the optimal CO_2_/N_2_ selectivity for PI-based MMM composites was observed at 20 wt % filler loading for MIL-101­(Cr) and [C_4_mim]­[Br]/MIL-101­(Cr), reaching a CO_2_ permeability of 9.9 Barrer and a CO_2_/N_2_ selectivity of 26.9. In contrast, 10 and 30 wt % MOF loadings resulted in selectivities of 15.2 and 19.7, respectively, highlighting the presence of an optimal filler content (see Table 2).

Another example employing “ship-in-a-bottle” immobilization method, was demonstrated by Iqbal et al.,^80^ who investigated the effect of a different MOF filler, UiO-66, along with a 3-(trimethoxysilyl)­propan-1-aminium acetate ([APTMS]­[Ac]) IL immobilized within its structure. Similar to the studies by Yasmeen and Ferreira, the MOF and IL were immobilized in a polymer matrix (poly­(ether–block–amide) (Pebax)—without the additional porous support, as graphically presented in Figure 6. Key findings revealed the uniform filler dispersion and a strong synergistic effect between the MOF and IL, leading to improved CO_2_ permeability while maintaining high mixed gases selectivity. The amount of UiO-66 filler was found to significantly influence membrane performance. Under mixed-gas conditions, the membrane with 20 wt % UiO-66 achieved a CO_2_ permeability of 143 Barrer, with CO_2_/N_2_ and CO_2_/CH_4_ selectivities of 55 and 25.4, respectively (see Table 2, entry 7). In comparison, the membrane with 30 wt % UiO-66 exhibited a slightly lower CO_2_ permeability of 98.3 Barrer, but higher CO_2_/N_2_ and CO_2_/CH_4_ selectivities of 61.1 and 28.3, approaching Robeson’s upper bound. The use of UiO-66, a stable, zirconium-based MOF, together with halide-free IL offered a sustainable solution for CO_2_ separation, in terms of environmental safety and compliance. However, further evaluation on membrane durability and potential recyclability is needed to assess its viability for scalable CO_2_ separation.

**Figure 6:**
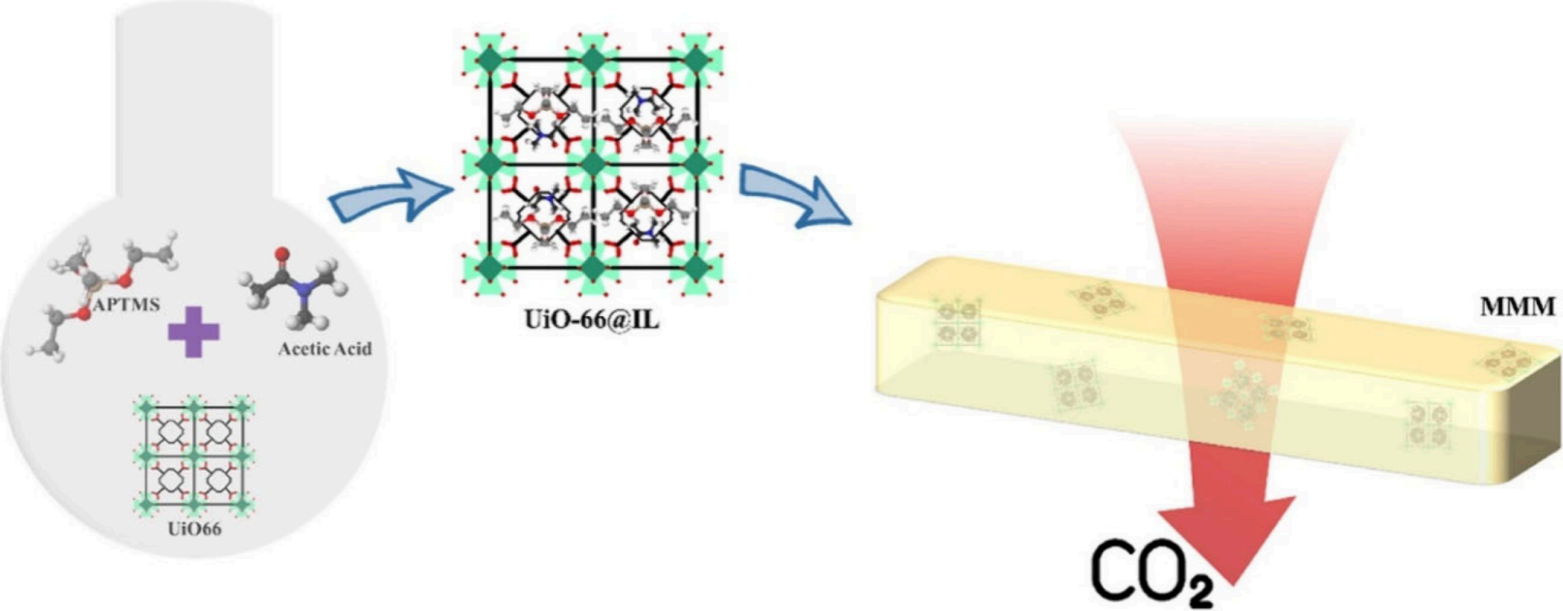
Scheme of MOF synthesis, IL’s immobilization and formed MMM. Reproduced from ref 80 with permission from Chemosphere. Copyright (2022) Elsevier.

### Graphene Oxide

Graphene-based materials, particularly GOs, have emerged as promising fillers for MMMs in TFC gas separation.^10^ GO, a two-dimensional allotrope of carbon, exhibits nanoscale thickness, exceptional surface area, and high mechanical strength. Special emphasis is placed on functionalizing GO to improve membrane/CO_2_ interactions and increase its permeation. Tenable transport channels, illustrated in Figure 7, can form between its layers and may function as molecular sieves, significantly improving membrane performance when its pore structure and layer stacking are precisely adjusted.^81–83^ However, detailed insights into the transport mechanisms in IL-GO MMMs remain limited, warranting further investigation.^84^

**Figure 7:**
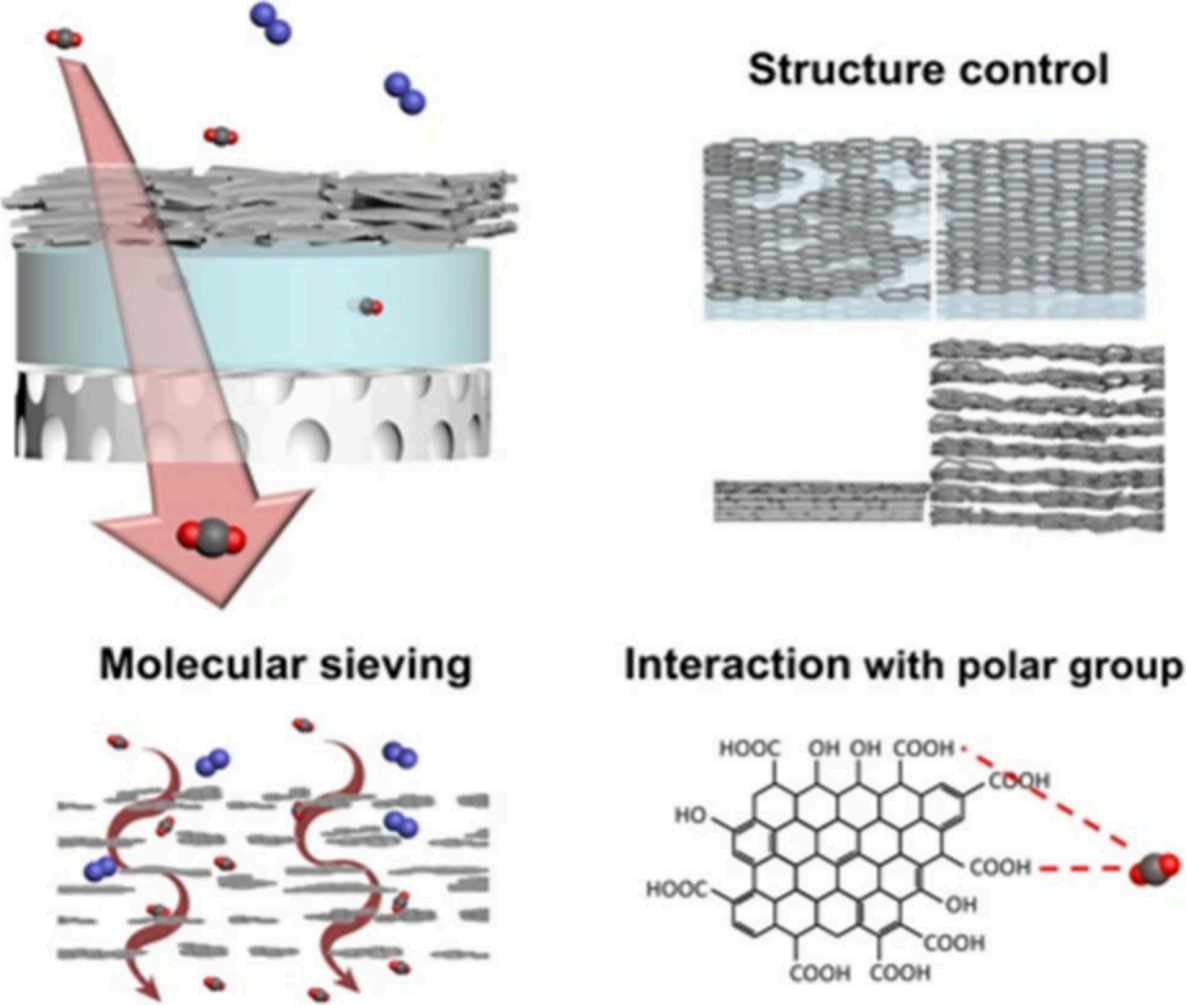
CO_2_ separation mechanisms in GO-modified membranes. Reproduced from ref 83 with permission from Sci. Rep. Copyright (2017) Nature Portfolio.

A supported membrane system (SILM) incorporating GO and ILs was developed by Karunakaran et al.^85^ TFC consisted of 20 wt % either 1-ethyl-3-methylimidazolium acetate ([C_2_mim]­[Ac]) or 1-ethyl-3-methylimidazolium tetrafluoroborate ([C_2_mim]­[BF_4_]) ILs, various GO loadings, and a poly­(1-trimethylsilyl-1-propyne) (PTMSP), dip-coated onto a porous ultrafiltration polyacrylonitrile (PAN) support. GO was found to stack on the membrane’s surface, forming sieving nanochannels that improved gas separation. As given in Figure 8 and Table 2, the incorporation of GO, along with an unselective but highly permeable sealing layer PTMSP, notably improved the CO_2_/N_2_ selectivity. The optimal GO loading of 0.5 wt % allowed us to exceed the CO_2_/N_2_ selectivities of 130 (for [C_2_mim]­[Ac]), proving the GO positive influence on membrane performance.

**Figure 8:**
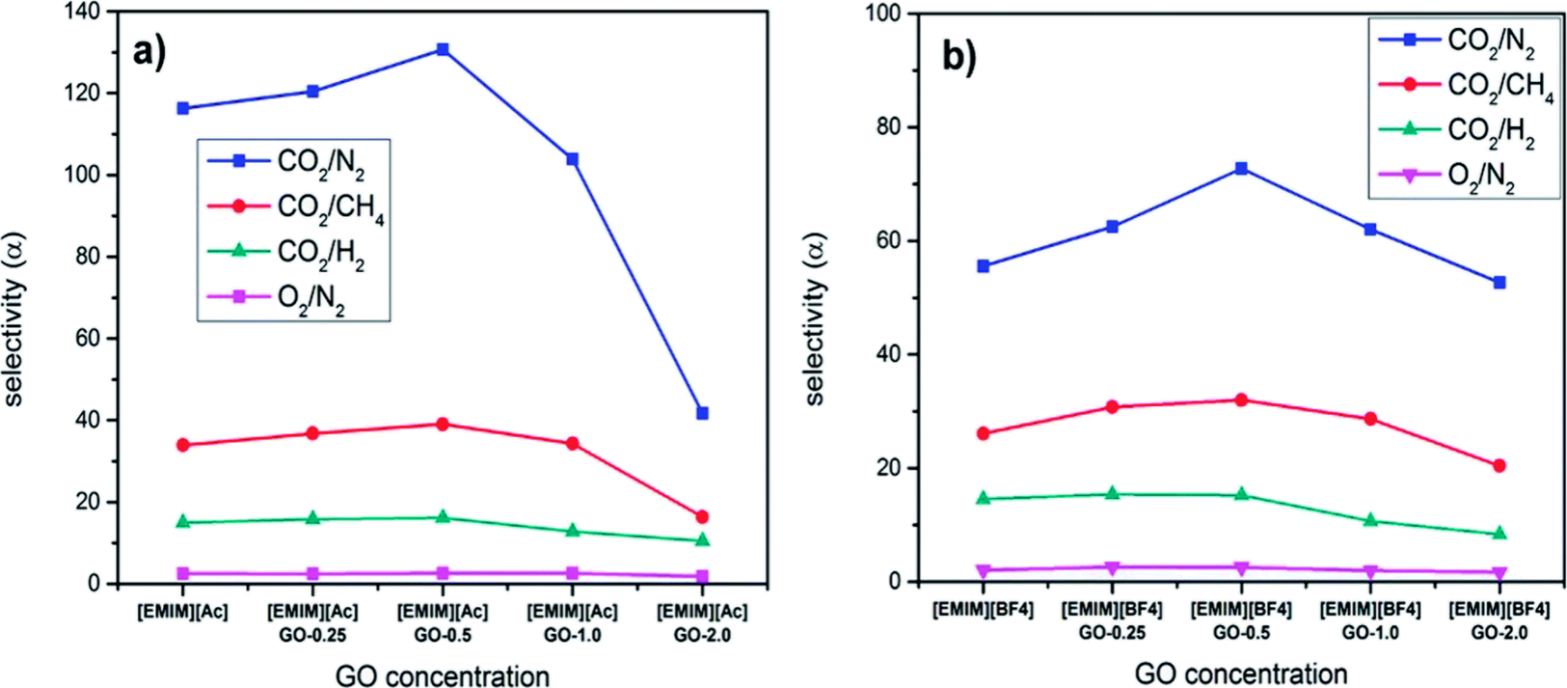
Influence of GO addition on selectivity, measured for [C_2_mim]­[Ac] and (b) [C_2_mim]­[BF_4_] membranes. Reproduced from ref 85 with permission from J. Mater. Chem. A. Copyright (2017) Royal Society of Chemistry.

In 2018, Huang et al.^86^ covalently functionalized GO with the IL 1-(3-aminopropyl)-3-methylimidazolium bromide ([APmim]­[Br]**-**NH_2_) and incorporated it into a Pebax matrix, later distributed over a polyvinylidene fluoride (PVDF) support. GO’s high-aspect-ratio sheets created a longer, more tortuous diffusion path, enhancing diffusivity selectivity. [APmim]­[Br]**-**NH_2_ further improved CO_2_ solubility and selectivity via reversible chemical interactions. Strong H-bonding between IL, GO, and Pebax ensured a uniform dispersion and enhanced polymer–filler compatibility. As given in Table 2, compared to neat Pebax, MMMs modified with IL and 0.05 wt % GO improved CO_2_/N_2_ selectivity by 90% and CO_2_ permeance by 50%, with CO_2_ permeance up to 900 GPU and CO_2_/N_2_ and CO_2_/H_2_ selectivities of ∼45 and 5.8, respectively. However, the further addition of GO resulted in a decline in both the CO_2_ permeance and selectivity, as shown in Table 2 and Figure 8. For optimal GO content (0.05 wt %) membranes surpassed 2008 Robeson’s upper bound, supporting their potential for practical CO_2_ separation and scale-up.

The application of graphene-based materials combined with PILs, used as fixed carriers for CO_2_ separation in was investigated by Lee et al.^87^ The membrane featured a selective layer of PIL-IL-rich gel, consisting of 1-ethyl-3-methylimidazolium 2-cyanopyridinium ([C_2_C_1_mim]­[2-CNpyr]) IL and polymerized diallyldimethylammonium 2-cyanopyrrolide (poly­[DADMA]­[2-CNpyr]), immobilized within a GO nanosheet framework, distributed over poly­(ether sulfone) (PES)/polyethylene terephthalate (PET) substrates. The incorporation of GO and its interactions with PIL/IL helped suppress IL leaching, while the PIL fixed carriers facilitated transport of CO_2_ across the membrane, as illustrated in Figure 9. Under direct air capture (DAC) conditions, the membrane achieved a CO_2_ permeance of 3090 GPU and a CO_2_/N_2_ selectivity of 1180, making it the highest-performing facilitated transport membrane to date.

**Figure 9:**
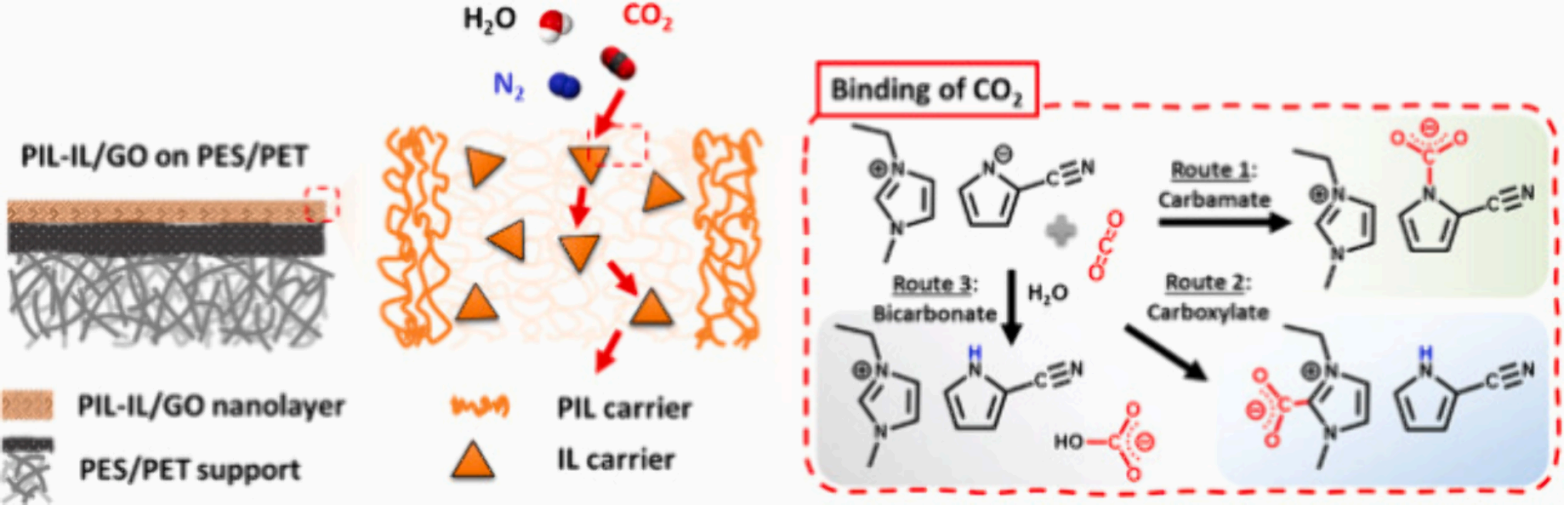
Facilitated transport membrane scheme and mechanism of binding of CO_2_ binding. Reproduced from ref 87 with permission from J. Membr. Sci. Copyright (2021) Elsevier.

Recently, Aghajohari et al.^88^ reported the use of interfacial polymerization (IP) to synthesize a TFC membrane incorporating nitrogen-doped GO (N-GO) and dicationic imidazolium ILs with trimethylene (C3IL) or hexamethylene (C6IL) linkers, confined within a polyamide (PA) layer on a PSF support. The N-GO nanosheets formed sieving channels and ultrasmall holes, improving the membrane performance. The addition of C3IL/N-GO enhanced CO_2_ permeance to 130 GPU, while CO_2_/CH_4_ and CO_2_/N_2_ selectivities reached 34 and 47, respectively. For C6IL/N-GO, the CO_2_ permeance decreased slightly to 100 GPU but remained high, while the CO_2_/CH_4_ and CO_2_/N_2_ selectivities improved to 36 and 48, respectively (see Table 3). These enhancements were attributed to ILs facilitating the uniform distribution of N-GO and promoting the formation of the CO_2_-accessible pores. Additionally, IL leaching was minimized through π-π interactions with N-GO, ensuring long-term stability.

**Table 3:** Comparison of Gas Permeation Results (Gas Permeances (P) and Selectivities (α)): Hollow Fiber Membranes^a^

No.	Membrane Description	M/S	P_feed_ (bar)	PCO_2_ (GPU)	PN_2_ (GPU)	αCO_2_/N_2_ (−)	ref
1	Pebax 1657/[C_2_mim][BF_4_]; IL: 80 wt %	S	3	300	8.3	36	^98^
							
2	Pebax/[C_6_mim][NTF_2_]	S	2	7.7	0.4	18.4	^99^
	Pebax/[C_6_mim][NTF_2_], IL: 10 wt %			19.3	2.7	7.1	
	Pebax/[C_6_mim][NTF_2_], IL: 20 wt %			17.5	2.4	7.4	
	Pebax/[C_6_mim][NTF_2_], IL: 40 wt %			23.3	2.7	8.7	
	Pebax/[C_6_mim][Cl], IL: 40 wt %			12.1	1.6	7.4	
							
3	Poly([C_2_vC_2_][Gly])	M	0.1	1400	1.4*	2000	^100^
							
4	Pure wSBC	S	3	245	250	1	^101^
	poly(S-IL); 1 wt % initiator: 1 wt %; polymerization t: 30 min			3.6	0.1	29	
							
5	poly(S-IL)	S	3	3.5	0.1	30	^103^
	poly(S-IL)/ZIF-8; ZIF-8:0.5 wt %			6	0.2	30	
	poly(S-IL)/ZIF-8; ZIF-8:1 wt %			5	0.2	28	
	poly(S-IL)/ZIF-8; ZIF-8:5 wt %			4	0.1	28	
							
6	Pebax/[C_2_mim][BF_4_]/GO; pH = 6	M	3	642	22.1	29	^105^
	Pebax/[C_2_mim][BF_4_] GO; pH = 12			981	22.3	44	

a Gas permeabilities are expressed in GPU; for reference, 1 GPU corresponds to 3.35 × 10^–10^ mol m^–1^ s^–1^ Pa^–1^, which is equivalent to 1 Barrer = 10^–10^ cm^3^(STP) cm cm^–2^ s^–1^ cmHg^–1^. Definitions: P_feed_, driving pressure difference; S/M, single or mixed gas conditions; *, data estimated for the comparison, not given in the article; [C_2_mim]­[BF_4_], 1-ethyl-3-methyimidazolium tetrafluoroborate; poly­([C_2_vC_2_]­[Gly]), poly­(vinylethylimidazolium glycine); wSBC, waste plastic styrene–butadiene copolymer; poly­(S-IL), poly­(styrene-based IL); GO, graphene oxide

Most recent studies have focused on the nanoconfinement of ionic liquids (ILs) within graphene oxide (GO) layers to achieve high-performance CO_2_ separation. For example, Dong et al. intercalated various imidazolium-based ILs into GO nanosheets to form nanoconfined IL membranes (GO-ILMs), which exhibited abnormal CO_2_ preferential permeation relative to smaller H_2_ molecules.^89^ The confinement enhanced CO_2_ solubility and diffusivity while blocking H_2_ transport, resulting in peak CO_2_ permeances of 13.85 GPU and CO_2_/H_2_ selectivity up to 13.58, surpassing the Robeson upper bound. Similarly, Behera et al. designed a GO/CNT hybrid network to spatially confine [EMIM]­[BF_4_], achieving a stable, ultrathin (∼230 nm) IL layer that combined a high CO_2_ affinity with fast nanochannel transport. This approach yielded a CO_2_ permeance of ∼600 GPU and CO_2_/N_2_ selectivity of 62 under humid and elevated-temperature conditions, demonstrating the advantage of nanochannel confinement and hybrid network design for scalable, high-performance membranes.^90^ Together, these studies highlight that nanoconfinement of ILs within GO-based architectures is a highly promising strategy to optimize both the selectivity and permeability for CO_2_ separation.

To facilitate comparison of the results summarized in Table 2, the data are presented in a Robeson-style plot (Figure 10), showing the CO_2_/N_2_ selectivity of the best-performing sample from each study as a function of the CO_2_ permeability (in GPU). Entries for which either CO_2_/N_2_ selectivity or CO_2_ permeability was not reported have been omitted. Each point in the plot is labeled with the corresponding entry in Table 2.

**Figure 10:**
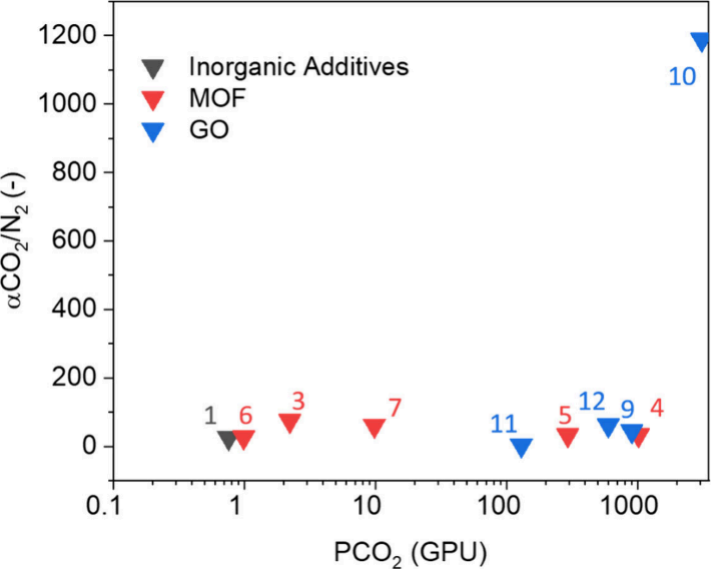
Robeson-style plot of the CO_2_/N_2_ selectivity versus the CO_2_ permeability (GPU) for the best-performing samples in each study. Each point is labeled with the corresponding entry in Table 2.

### Hollow Fiber Membranes

As an alternative to exploring different materials for membrane selective layers, another promising research direction that should not be overlooked when discussing ways to enhance the performance of IL-functionalized polymeric membranes is the optimization of membrane module configurations. It is important to highlight that this approach differs from the previously discussed chemical composition-oriented IL membrane designs, as it focuses on membrane geometry and engineering rather than chemical functionality. Among these, hollow fiber membranes (HMF) provide a more efficient alternative to conventional flat-sheet membranes or spiral-wound geometries. As presented in Figure 11, hollow fibers offer a significantly higher surface area-to-volume ratio due to their cylindrical geometry and smaller diameter, which allows for a compact module design with a much higher membrane packing density. This significant geometrical efficiency translates into increased permeation flux, better utilization of module space and reduced carbon footprint, which all contribute significantly to their overall attractiveness for scaling up in industrial industry.^91–95^

**Figure 11:**
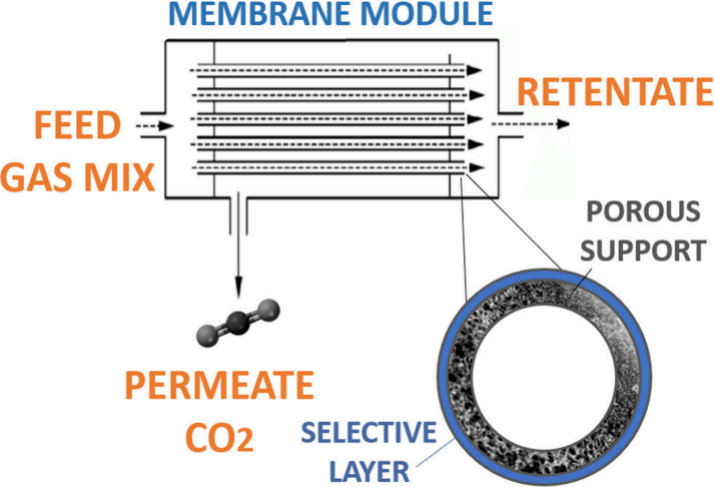
Schematic representation of hollow fiber membrane module and single fiber cross-section. Adapted from ref 99 with permission from ACS Sustain. Chem. Eng. Copyright (2024) American Chemical Society.

Despite their numerous advantages, the fabrication of high-quality hollow fibers, along with their functionalization (e.g., via coating methods) and long-term application, remains a challenge compared with the production of flat sheet membranes, particularly at the laboratory scale. Due to their thin walls and small diameters, hollow fibers can exhibit reduced mechanical stability, especially during module assembly or under high-pressure operation. Properly sealing and potting large bundles of fibers is a nontrivial task, and inadequate fabrication quality can result in leaks or diminished membrane performance. Moreover, hollow fibers are prone to the formation of macrovoids and pinholes, defects that can substantially deteriorate membrane performance over extended use. Process scalability and reproducibility also present challenges, particularly in achieving uniform wall and coating thickness, consistent morphology, and reliable performance across batches.^91,96,97^ While these limitations are balanced by the considerable advantages of hollow fiber membranes, they highlight important technical barriers in fabrication and application. The following examples of research on IL-functionalized HFMs describe recent strategies to address these challenges for CO_2_ separation.

In 2017 Fam et al.^98^ fabricated a HFM composite, functionalized with Pebax/IL blend. PVDF fibers were dip-coated with a selective Pebax layer containing up to 80 wt % [C_2_mim]­[BF_4_]. A polymeric (PTMSP) thin gutter layer was applied to prevent potential IL migration. The IL preferentially interacted with Pebax’s poly­(ethylene oxide) (PEO) segments via hydrogen bonding, reducing polymer crystallinity. However, this interaction limited CO_2_ binding, lowering the gas permeability below the expected values. As given in Table 3, the membrane achieved a CO_2_ permeance of 300 GPU, with CO_2_/N_2_ and CH_4_/N_2_ selectivities reaching 36 and 15, respectively. Long-term performance was declined due to PTMSP aging, causing a 40% permeance decrease over seven months, highlighting the importance of coating optimization. Future work could focus on enhancing durability and investigating membrane performance under high-humidity and saturated conditions to better understand the operational stability.

Piotrowska et al.^99^ recently optimized the fabrication of Pebax/IL-functionalized HFMs, employing a novel continuous-coating method to achieve uniform coverage of polypropylene (PP) hollow fibers, demonstrating its strong potential for industrial applications. The resulting hollow fiber composites, coated with Pebax and varying amounts of two imidazolium-based ILs—[C_6_mim]­[NTf_2_] or [C_6_mim]­[Cl]—exhibited mechanical pressure stability up to 5 bar, confirming their suitability for industrial gas separation. While the incorporation of IL resulted in a decrease of selectivity, it simultaneously enhanced the CO_2_ permeability. Pebax-based coatings with [C_6_mim]­[NTf_2_] (40 wt %) achieved the best gas separation performance (exact values in Table 3). The hollow fibers reached a CO_2_ permeance of 23.3 GPU and a CO_2_/N_2_ ideal selectivity of 8.7. The CO_2_/CO ideal selectivity reached 12.4, indicating the potential for applications targeting these gas separations. The authors noted that further coating optimization, such as multilayer structures, could improve performance, and that detailed comparisons with existing technologies would provide additional context for future work.

Kamio et al.^100^ proposed an alternative coating strategy, creating a facilitated transport HFM by coating the inner wall of PSF hollow fibers. In contrast to the aforementioned studies that blended ILs with polymer matrices, their approach utilized polymerized ILs in the form of poly­(vinylethylimidazolium amino acid) (poly­([C_2_vC_2_]­[AA]) gel particles. These gel particles were functionalized with glycine (Gly), serving as a CO_2_ carrier to enable facilitated transport. For membrane fabrication, the PSF support was placed in a gas permeation (GP) cell, sealed at both ends, and filled with an aqueous suspension of the gel particles. Using nitrogen flow and constant N_2_ pressure, a uniform ∼1 μm-thick gel layer was successfully deposited onto the inner surface of the PSF support through a filtration-based coating process (see Figure 12).

**Figure 12:**
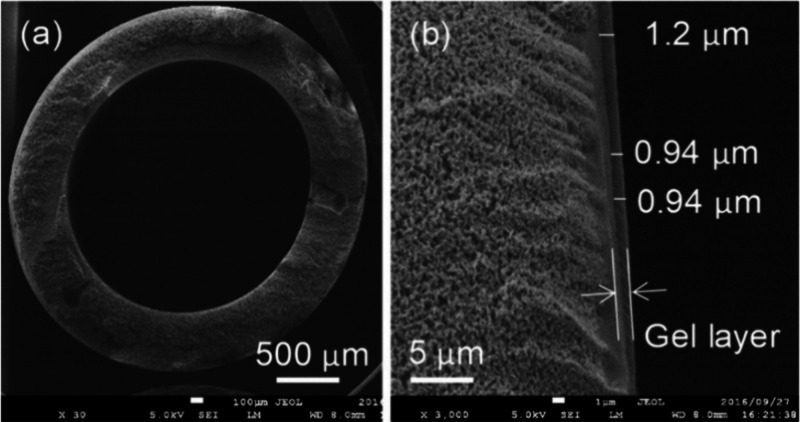
SEM images of the coated HFM cross section (a) and magnification on the coated inner gel layzer (b). Reproduced from ref 100 with permission from Ind. Eng. Chem. Res. Copyright (2020) American Chemical Society.

As shown in Table 3, the membrane exhibited high CO_2_ permeance, benefiting from the facilitated transport mechanism, attributed to the addition of glycine – natural amino acid which can reversibly react with CO_2_ through its amine group. Under mixed gas and humid (80%) conditions, it achieved a CO_2_ permeance of 1400 GPU and a CO_2_/N_2_ selectivity above 2000, making it highly promising for more sustainable direct air capture applications.

Another notable study on PIL-based coatings for HFMs was proposed by Lai et al.,^101^ who introduced a novel UV photopolymerization approach to fabricate the selective layer. In this method, a poly­(styrene-based IL) (poly­(S-IL)) was UV photopolymerized in situ on the external surface of hollow fibers, forming a stable selective layer. The support material, waste plastic styrene–butadiene copolymer (wSBC) was previously reported for its high permselectivity and excellent transmembrane pressure stability.^102^ Photopolymerization parameters significantly influenced the membrane performance. Higher precursor concentrations and longer polymerization times resulted in defect-free membranes with improved CO_2_/N_2_ selectivity, while increasing the UV initiator concentration led to a denser structure, reducing the CO_2_ permeance. The optimal membrane, prepared with a 30 min polymerization and 1 wt % photoinitiator, achieved a CO_2_ permeance of 3.6 GPU and a CO_2_/N_2_ selectivity of 29 (Table 3). Additionally, higher operating temperatures enhanced CO_2_ transport, increasing selectivity by 28.95% compared to single gas tests, demonstrating the membrane’s potential under realistic conditions. Importantly, the use of recycled waste plastic as membrane support combined with solvent-free UV curing underscores the method’s sustainability and suitability for eco-friendly, scalable industrial membrane production.

As the continuation of their prior research, Lai and colleagues^103^ further tried to enhance CO_2_ permeance of poly­(S-IL)-based HFMs by incorporating ZIF-8 NPs, which may serve as molecular sieves. They also investigated the impact of the SIL monomer’s alkyl chain length. GP results showed that shorter alkyl chains enhanced the CO_2_ permeance (from 3.6 to 4.6 GPU) but decreased the CO_2_/N_2_ selectivity (from 29 to 23). Adding 3.4 Å ZIF-8 NPs improved the CO_2_ permeance by 33% (from 3.6 to 4.8 GPU) without sacrificing the CO_2_/N_2_ selectivity. However, further increases in the ZIF-8 content led to chain rigidification and reduced the level of CO_2_ permeance. Screening of the additive content (see Table 3) revealed that an optimal ZIF-8 loading of 0.5 wt % resulted in a CO_2_ permeance of 6 GPU and a CO_2_/N_2_ selectivity of 30. The interaction between ZIF-8 and the membrane, including π-π interactions, enhanced compatibility, creating a complete membrane structure.

As discussed in previous chapters, the incorporation of GO can significantly enhance the membrane separation performance. Fam et al.^104^ combined the advantages of PVDF HF morphology together with mixed matrix-based coating, containing GO and IL – 1-ethyl-3-methyimidazolium tetrafluoroborate ([C_2_mim]­[BF_4_]). GO addition up to 0.5 wt % enhanced the CO_2_ permeance while maintaining the CO_2_/N_2_ selectivity. Higher loadings led to GO aggregation and reduced permeability. GO influenced IL behavior by increasing migration, weakening IL/Pebax interactions, and causing microphase separation. As shown in Table 3, entry 6, alkaline conditions enhanced both the CO_2_ permeance and the CO_2_/N_2_ selectivity due to accelerated IL migration and strengthened interactions with CO_2_. High permeance and stability in humid, NO_x_-containing streams improved process efficiency and durability, supporting eco-friendly CO_2_ capture and advancing CCU strategies under real industrial conditions.

In addition to the aforementioned examples of relevant studies on hollow fiber membranes, it is important to emphasize that HFMs require particular attention for long-term stability and durability, as their thin walls and high packing densities make them more susceptible to mechanical deformation, wetting, and performance decay under realistic operating conditions. Several studies have evaluated the long-term behavior of IL-based or IL-compatible HFMs under continuous operation.

As an example, evidence of the robustness of HFM systems under extended operation was provided by Pang et al., who investigated hybrid PVDF–SiO_2_–HDTMS hollow fibers for CO_2_ absorption. The membranes exhibited superhydrophobic outer surfaces and enhanced mechanical strength due to inorganic nanoparticle incorporation. Under continuous operation for 20 days in a gas–liquid contacting CO_2_ absorption process (CO_2_/N_2_ = 19/81), the CO_2_ mass-transfer flux decreased by only 3%, highlighting the importance of surface hydrophobicity and structural reinforcement for suppressing pore wetting and maintaining long-term performance.^106^ These studies demonstrate that with appropriate material design, HFMs can achieve the long-term mechanical stability and operational durability required for industrial CO_2_ separation applications.

The presented examples indicate that the optimization of IL-functionalized polymeric composite membranes continues to be an active area of research. Advances such as refinement of PILs, incorporation of MOFs and GOs into MMMs, and improvements in module configurations and coating techniques have contributed to enhanced CO_2_ separation performance. These developments span both material-level innovations and engineering-level optimization (e.g., hollow fiber membrane configuration), underscoring the multifaceted nature of progress in IL-based membrane technologies. Nevertheless, each study also highlighted limitations and areas for further improvement, providing guidance for the continued development of membrane efficiency and industrial applicability. Comprehensive comparisons with existing technologies would further clarify the practical potential of these membranes.

## Dual Function of Ionic Liquid-Functionalized Membranes: CO_2_ Separation and Conversion

As previously stated, ILs enhance the CO_2_ conversion through multiple synergistic mechanisms that improve the reaction efficiency, selectivity, and stability. Their strong affinity toward CO_2_ arises from both physical absorption and specific chemical interactions – such as Lewis acid–base binding – which increase CO_2_ solubility and facilitate activation. They can be widely applied in various CO_2_ valorization pathways, including the use as cocatalysts in transition metal-based catalysis, enzymatic CO_2_ conversion, photo- or photoelectrocatalysis, as well as electrocatalysis.^107–112^

In these systems, ILs play a crucial role by stabilizing metal catalysts, enhancing gas solubility, and facilitating molecular interactions that improve the overall reaction efficiency. The integration of ionic liquid-promoted catalytic CO_2_ conversion and CO_2_ separation can be realized using a membrane reactor system, where membrane-based separation and catalysis occur simultaneously. This approach enables us to effectively shift the equilibrium of reaction in the desired direction by continuous removal of the product. The key advantages of membrane reactors include reduced separation costs and the ability to purify fewer stable substances, along with the suppression of unwanted side reactions, resulting in enhanced reaction selectivity. Additionally, catalyst lifetimes can be extended through efficient catalyst separation and recycling. Moreover, multiple membranes can be connected in series to facilitate consecutive reaction steps within a single device.^113–115^ Successful implementation of ILs in catalytic membrane reactors has been already reported in the literature, comprising various reactions, which are not limited to CO_2_ conversion only, namely esterification, water gas shift or Suzuki-Miyaura reaction.^116,117^

These synergistic effects arise from the interplay between IL-mediated CO_2_ activation and membrane-enabled separation. ILs not only increase the CO_2_ solubility and facilitate chemical activation through specific interactions with the substrate but also stabilize catalysts and intermediates, enhancing reaction rates and selectivity. When combined with membrane separation, the continuous removal of products shifts reaction equilibria toward desired products, suppresses side reactions, and enables more efficient use of the catalyst. Together, these mechanisms create a mutually reinforcing system in which CO_2_ conversion and separation performance are enhanced simultaneously.^118,119^

An emerging research direction in this field utilizes kinetic modeling, to enhance simultaneous CO_2_ conversion and product removal. In 2025, Sun et al.^120^ applied this approach to predict the performance of a porous IL membrane reactor for the selective conversion propylene oxide (PO) with CO_2_ into polycarbonate (PC), under nonisothermal condition. A mathematical model of a porous IL membrane reactor was developed to analyze the mass transfer and reaction kinetics. The study explored IL membrane layers as Newtonian and non-Newtonian fluids, examining flow velocity distributions in plate and tubular configurations. The reaction kinetics, based on the Arrhenius equation, showed an activation energy of 14.70 kJ/mol, though the PO conversion rate was 9.04%, lower than the theoretical predictions, suggesting the potential for further mass transfer enhancements. A concentration distribution model was established, achieving 86% PO to PC conversion with 81.26% PO decomposition. While the model demonstrated the potential of IL membrane reactors for CO_2_-epoxide conversion, IL loss was noted as a key issue affecting long-term stability. Overall, IL incorporation improved the CO_2_-epoxide interactions, reduced the activation energy, and enhanced the reaction rates. The developed model provides a useful framework for predicting reaction kinetics and reactor performance in IL-based catalytic membrane systems.

The experimental synthesis of PC from CO_2_ and PO via cycloaddition was demonstrated in 2022 by the group of Sun et al.,^121^ where they developed a novel catalytic membrane reactor for the PC formation, operating under continuous flow conditions without the need for additional solvents or cocatalysts. The membrane support consisted of tubular ceramic alumina (α-Al_2_O_3_), thermally modified, with a mesoporous γ-Al_2_O_3_ top layer featuring 2.67 nm pores. As illustrated in Figure 13., the catalytic membrane was formed by encapsulating butyltriphenylphosphonium bromide ([PPh_2_Bu]­Br) IL within polyvinylpyrrolidone (PVP), which based on its high viscosity served as both a binder and membrane-forming agent. The IL-immobilized PVP layer was thermally coated onto the γ-Al_2_O_3_ nanochannels, creating a dense 2.98 μm-size-range catalytic layer over a 15.7 μm intermediate layer. The system achieved efficient CO_2_ cycloaddition at 80 °C with PVP playing a crucial role in inhibiting IL loss and stabilizing the catalytic layer. At 120 °C, complete PO conversion and 100% PC selectivity were observed, while at lower temperatures, the PC yield reached a minimum of 46.93%. The high conversion and selectivity demonstrated the feasibility of this approach with potential expansion toward direct CO_2_ capture from flue gas into ILs for in situ cyclic carbonate formation. However, achieving this would require chemical grafting of ILs onto the support to enhance long-term stability.

**Figure 13:**
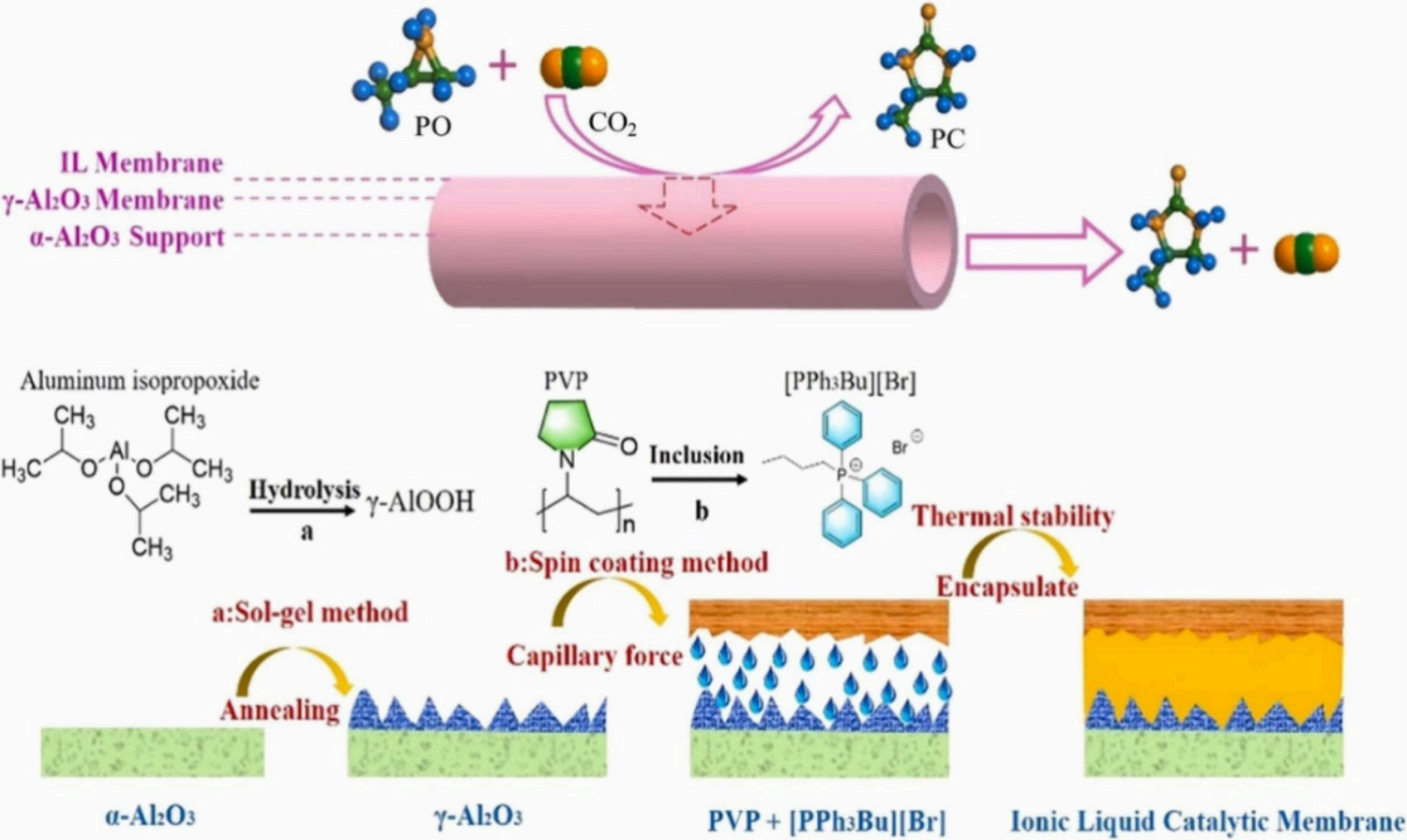
Graphical representation of the supported ionic liquid membrane catalyzing the CO_2_ cycloaddition. Schematic representation of supported, IL-functionalized membrane (top) and IL-based catalytic membrane preparation (bottom), reproduced from ref 121 with permission from J. CO_2_ Util. Copyright (2018) Elsevier.

Another example of reactions that explore the potential of polymeric composite membranes incorporating ILs is the water gas shift (WGS). This reaction primarily focuses on converting CO and H_2_O into CO_2_ and H_2_, rather than directly capturing, separating and converting CO_2_. However, if the CO_2_ generated from the WGS is immediately separated and converted within the same IL-functionalized membrane system, it becomes part of an integrated CO_2_ valorization strategy.

In the studies published by the group of Nancarrow^122^ flat-sheet PI membranes were functionalized with ILs—1-butyl-3-methylimidazolium triflate ([C_4_mim]­[OTf]) or [C_4_mim]­[NTf_2_]—along with a dissolved Ru carbonyl complex (RuCl_3_·xH_2_O) to facilitate the WGS reaction by simultaneous CO content reduction and enhanced H_2_/CO separation efficiency. The study aimed to optimize the membrane configurations by varying the IL anion type and content, catalyst concentration, temperature, pressure, and time-on-stream. It was found that increasing the concentration of [C_4_mim]­[OTf] and RuCl_3_·xH_2_O improved the turnover frequencies (TOFs). However, excessive RuCl_3_·xH_2_O reduced the membrane mechanical strength, while higher IL concentrations led to Ru complex evaporation. The optimal conditions were determined to be 2 wt % RuCl_3_·xH_2_O, 20 wt % [C_4_mim]­[OTf], 140 °C, and 2 bar, offering a balance between reactivity and durability. The study also demonstrated that the water–gas shift reaction effectively reduced the CO concentration and increased H_2_/CO selectivity from 15.7 to 36.9, proving its potential for future applications.

Another example of membrane reactor which utilized ILs for the catalysis of low-temperature WGS was developed by Logemann et al.^123^ They presented a catalytic system that contained a Ru-based catalyst, specifically [Ru­(CO)_3_Cl_2_]_2_, dissolved in the IL 1-butyl-2,3-dimethylimidazolium chloride ([C_4_C_1_C_1_Im]­Cl). The catalyst-IL mixture was supported on alumina pellets, which were placed inside the channels of a ceramic silicon carbide (SiC) monolith (Figure 14). To enhance gas separation, the monolith was coated with a polydimethylsiloxane (PDMS) gutter layer and a polymeric gas separation coating (from a mixture of poly­(vinyl alcohol) (PVA) and poly­(vinylamine) (PVAm), enabling CO_2_ removal via a facilitated transport mechanism. This selective CO_2_ separation was designed to shift the reaction equilibrium toward H_2_ formation. The study demonstrated that the membrane exhibited optimal gas separation performance under humidified conditions, as water swelling increased the CO_2_/N_2_ selectivity by 10–15 times. However, the CO_2_/H_2_ selectivity was limited to 2.1, attributed to low humidity levels due to reactor design constraints. The catalytic system showed high activity at 160 °C, aligning with low-temperature WGS conditions, and remarkable stability over 240 h-operation. The use of nonvolatile IL media, low-temperature operation, and extended catalyst stability further supported the system’s alignment with green chemistry and energy-efficient homogeneous WGS catalysis and in situ CO_2_ separation.

**Figure 14:**
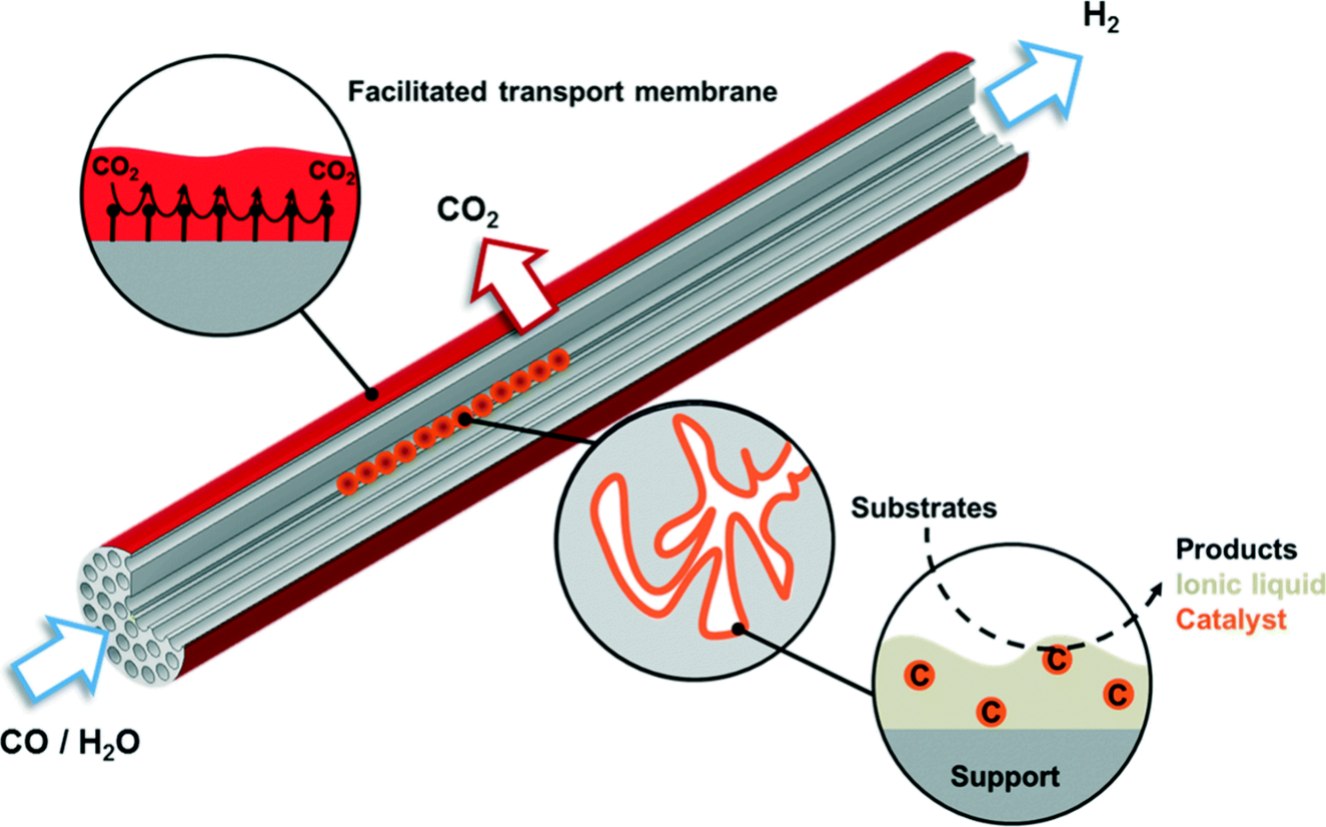
Graphical representation of the supported IL-catalytic system inside the SiC monolith and polymeric FTM on top. Reproduced from ref 123 with permission from Green Chem. Copyright (2021) Royal Society of Chemistry.

Finally, the simultaneous CO_2_ capture and conversion in autonomous integrated system were presented by Vishwakarma et al.^124^ In contrast with the previously discussed studies, ILs were not immobilized within the membrane. In this system, 1,8-diazabicyclo[5.4.0]­undec-7-ene (DBU)-based ILs were immobilized on superamphiphobic silicon nanowire (SiNW) bundles, facilitating CO_2_ fixation and subsequent conversion. The system operated with a stable gas–liquid interface maintained by the superamphiphobic SiNWs, with the liquid flow of amines in the upper part of the channel and the gas flow of CO_2_ in the bottom part. The CO_2_ gas–liquid reactions were carried out in a laminar flow, where CO_2_ was captured and converted on-site. The reaction involved liquid amines reacting with CO_2_ to produce 2-oxazolidinones and quinazoline-2,4­(1H,3H)-diones, achieving a yield of 81–97% under mild conditions. This approach was extended to natural gas containing 2.95% CO_2_ (simulating flue gas), demonstrating its potential for direct air capture and natural gas purification. Although this study does not employ an IL-functionalized polymer-based separation membrane (a polymeric (PTFE) membrane was used separately for reaction workup – it still embodies the concept of simultaneous CO_2_ separation and catalysis. To provide a broader perspective on IL-enhanced systems for CO_2_ separation and valorization, the authors of this work have included this valuable research example.

Despite the promising examples of successful integration of IL-enhanced CO_2_ catalysis with membrane-based CO_2_ separation, finding comprehensive and representative studies of this combined approach remains challenging. Among the key difficulties in integrating these technologies, the most significant include the high viscosity of ILs, IL leaching during contact with water (either in the reaction or separation stages), which can lead to catalyst loss and product contamination,^125^ as well as IL-induced swelling of polymers, which can lead to membrane degradation or alterations in their pore structure With respect to the permeability–selectivity trade-off, ILs typically increase membrane permeability, which may result in a loss of selectivity.^99^ Additionally, while ILs exhibit high thermal stability, the decomposition temperature of the polymer is often the limiting factor, restricting the application of IL-functionalized membranes in high-temperature CO_2_ conversion processes.^126^ Overall, while the implementation of IL-functionalized polymeric membranes remains an ongoing challenge, the examples discussed above not only underscore the success of this approach but also offer insights into the emerging direction.

## Summary and Outlook

### Summary of Discussed Solutions and Current Challenges

Combining ILs with membrane technology has emerged as a highly promising strategy for mitigating the impact of CO_2_ emissions through efficient separation and catalytic conversion. In CO_2_ separation, the functionalization of polymer-based membranes with ILs can significantly enhance performance due to favorable IL – CO_2_ interactions that increase CO_2_ uptake. Recent research has explored advanced strategies to further improve IL-functionalized membranes, as discussed in this Review. Special emphasis has been placed on the synthesis and functionalization of polymerized ILs (PILs), the development of mixed matrix membranes (MMMs) incorporating inorganic salts, MOFs, and GO, and the optimization of membrane configurations, particularly hollow fiber membranes. The examples presented here demonstrate strong potential for improving CO_2_ permeability, selectivity, and, in some cases, operational stability in humid, NO_x_-containing streams, supporting sustainable CO_2_ capture and advancing CCU strategies under industrially relevant conditions. To fully exploit the high CO_2_/N_2_ selectivity, membranes must withstand high feed-to-permeate pressure ratios. Thus, long-term pressure stability, alongside chemical and thermal resistance, is crucial for industrial scale-up.^127^

Beyond separation, ILs also serve as effective promoters of CO_2_ valorization, functioning as solvents or (co)­catalysts. Membrane reactors incorporating ILs enable simultaneous catalytic conversion and separation, facilitating equilibrium shifts toward desired products while simplifying the process design. Reported examples include CO_2_ cycloaddition and the water–gas shift reaction, supported by both experimental demonstrations and kinetic modeling studies.

Despite these advances, several critical challenges hinder the practical deployment of IL-functionalized membranes. Phase incompatibility between ILs and polymer matrices can lead to aggregation, phase separation, or poor IL retention, degrading the membrane performance over time. The thermal stability and chemical robustness of the polymer support also place constraints on the operating conditions. Moreover, when operated under elevated transmembrane pressures, the IL may leach from supported membranes as capillary forces are insufficient to fully retain the IL phase. The classical permeability–selectivity trade-off remains unresolved in many IL-based membranes, and the majority of studies are carried out under idealized laboratory conditions. Real-world process streams (e.g., flue gas containing humidity, NO_x_, or particulate impurities) are much more complex and can further challenge membrane stability and performance.^128,129^

To translate laboratory advances into industrially relevant technologies, future research must focus on well-defined performance metrics or key performance indicators (KPIs) for each type of IL-functionalized membrane. High CO_2_ permeance is essential to reduce the membrane area and capital cost, while high CO_2_/N_2_ selectivity ensures product purity and minimizes downstream separation. Long-term stability under realistic conditions, including high feed-to-permeate pressure ratios, humidity, temperature cycling, and potential IL leaching, is critical, as are mechanical robustness and module durability, particularly for thin-film or supported membranes. For example, polymerized PILs typically target CO_2_ permeances of 50–500 GPU and selectivities of 20–50, with high thermal stability and resistance to IL leaching. MMMs with MOFs, graphene oxide, or salts can achieve 20–100% higher permeance and 10–30% higher selectivity than neat polymers while maintaining hundreds of hours of operational stability. Hollow fiber membranes provide high module packing densities and tolerate feed-to-permeate pressures of 1–5 bar, although long-term mechanical robustness remains a key consideration.^119,130^

### Future Opportunities

Apart from the advanced strategies discussed in this Review, several emerging directions hold strong potential to deliver the next major breakthroughs in IL-functionalized membranes. One particularly promising area is the development of dynamic covalent network polymers based on PIL chemistry including recently reported self-healing PIL membranes. These materials incorporate reversible covalent linkages and ionic clusters that enable structural regeneration under mild conditions, defect repair, and enhanced mechanical robustness under CO_2_-rich conditions, thus addressing the longstanding issues related to long-term stability and pressure resistance.^131–133^ IL-hydrogel composite membranes combine the favorable gas-solvation characteristics of ILs with the flexibility, tunable porosity, and significant water retention of hydrogel networks. Such hybrid soft-matter architectures may provide new opportunities for CO_2_ capture under humid conditions, where conventional polymeric membranes often suffer performance loss.^134,135^ In addition to new membrane materials, the field is moving toward responsive and adaptive systems. Photoresponsive ILs, whose physicochemical properties can be reversibly modulated by light, offer a pathway to externally controlled transport behavior and dynamic tuning of CO_2_ permeability or selectivity.^136,137^ Likewise, biomimetic channel design—drawing inspiration from the highly selective transport mechanisms of biological ion channels—may allow ILs or PILs to organize into ordered, subnanometer transport pathways that transcend the traditional permeability–selectivity trade-off.^138,139^ Finally, a relevant future direction is the development of eco-friendly and sustainable ILs, including bioderived cations, amino-acid-based anions, and low-toxicity, readily recyclable IL systems, which can reduce environmental burden while maintaining high CO_2_ affinity. Such approaches have already been demonstrated in emerging classes of biodegradable or biosourced ILs and PILs.^140,141^ Together, these emerging concepts suggest a shift toward ecological, smarter, more durable, and more efficient IL-based membrane platforms, offering exciting opportunities for future innovations in CO_2_ separation and catalytic membrane technologies.

Overall, IL-functionalized polymeric membranes represent a rapidly developing field with considerable potential. Importantly, current advances span both molecular-level IL design and engineering-level membrane configuration, highlighting the multidisciplinary nature of progress in this area. Continued developments in material design, membrane configuration, and reactor integration are expected to further improve the separation efficiency and catalytic functionality. To fully realize industrial implementation, future research should also emphasize scalable fabrication methods and techno-economic evaluation. Together, these advances provide a strong foundation for translating laboratory-scale findings into competitive membrane technologies for CO_2_ capture and utilization.

## Supplementary Material



## Notes

Structures of the ionic liquids given in Table 1 (PDF)

## Abbreviations

[APmim]­[Br]-NH_2_: 1-(3-aminopropyl)-3-methylimidazolium bromide
[APTMS]­[Ac]: 3-(trimethoxysilyl)­propan-1-aminium acetate
[BMIM]­[BF_4_]: 1-butyl-3-methylimidazolium tetrafluoroborate
[C_1_mim]­[BF_4_]: 1-butyl-3-methylidazolium tetrafluoroborate
[C_2_C_1_mim]­[2-CNpyr]: 1-ethyl-3-methylimidazolium 2-cyanopyridinium
[C_2_mim]­[Ac]: 1-ethyl-3-methylimidazolium acetate
[C_2_mim]­[BF_4_]: 1-ethyl-3-methylimidazolium tetrafluoroborate
[C_2_mim]­[NTf_2_]: 1-ethyl-3-methylimidazolium bis­(trifluoromethylsulfonyl)­imide
[C_3_mim]­[Br]: 1-propyl-3-methyl-imidazolium bromide
[C_3_PTMSam]­[Ac]: 3-(trimethoxysilyl) propan-1-aminium acetate
[C_4_C_1_C_1_Im]: Cl 1-butyl-2,3-dimethylimidazolium chloride
[C_4_mim]­[Br]: 1-butyl-3-methylimidazolium bromide
[C_4_mim]­[NTf_2_]: 1-butyl-3-methylimidazolium bis­(trifluoromethylsulfonyl)­imide
[C_4_mim]­[Otf]: 1-butyl-3-methylimidazolium triflate
[C_6_mim]­[Cl]: 1-hexyl-3-methylimidazolium chloride
[C_6_mim]­[NTf_2_]: 1-hexyl-3-methylimidazolium bis­(trifluoromethylsulfonyl)­imide
[C_6_mim]­[NTf_2_]: bis­(trifluoromethylsulfonyl)­imide
[P_66614_]­[Cl]: trihexyltetradecylphosphonium chloride
[Pyrr_14_]­[NTf_2_]: N-butyl-*N*-methyl pyrrolidinium bis­(trifluoromethylsulfonyl)­imide
Al_2_O_3_: alumina
AM: acrylamide
ATR-FTR: attenuated total reflectance - Fourier transform infrared spectroscopy
BA: butyl acrylate
C3IL: trimethylene linker
C6IL: hexamethylene linker
CCU: carbon capture and utilization
CO: carbon monoxide
CO_2_: carbon monoxide
COF: covalent organic framework
DAC: direct air capture
DBU: 1,8-diazabicyclo­[5.4.0]­undec-7-ene
Gly: glycine
GO: graphene oxide
GP: gas permeation
HFM: hollow fiber membrane
IL: ionic liquid
IP: interfacial polymerization
KPI: key performance indicators
LiTFSI: lithium bis­(trifluoromethanesulfonyl)­imide
MMM: mixed matrix membrane
MOF: metal organic frameworks
NPs: nanoparticles
PA: polyamide
PAM: polyacrylamide
PAN: polyacrylonitrile
PAP: poly­(1-allyl-3-methylimidazoliumbis­(trifluoromethanesulfonyl)­imide-co poly­(ethylene glycol) methyl ether methacrylate
PC: polycarbonate
PCMS: poly­(4-chloromethylstyrene)
PDMS: polydimethylsiloxane
Pebax: poly­(ether-*block*-amide)
PEO: poly­(ethylene oxide)
PES: poly­(ether sulfone)
PET: polyethylene terephthalate
PI: polyimide
PIL: poly­(ionic liquid)
PO: propylene oxide
poly­([C_2_amvtaz]­[Br]-*co*-PAm): poly­[1-(p-vinylbenzyl)-3-methylimidazolium glycinate]
P­([VBMI]­[Gly]): poly­(4- aminoethyl-1-vinyl-1,2,4-triazolium) bromide-*co*-polyacrylamide
poly­([C_2_vC_2_]­[AA]): poly­(vinylethylimidazolium amino acid)
poly­([C_2_vC_2_]­[Gly]): poly­(vinylethylimidazolium glycine) poly­([C_2_vC_2_]­[Gly]) poly­(vinylethylimidazolium glycine)
poly­([C_4_vim]­[Cl]): poly­(1-vinyl-3-butylimidazolium chloride)
poly­([C_4_vim]­[NTf_2_]): poly­(1-vinyl-3-butylimidazolium bis­(trifluoromethylsulfonyl)­imide)
poly­([DADMA]­[2-CNpyr]): poly diallyldimethylammonium 2-cyanopyrrolide
poly­([DADMA]­[NTF_2_]): poly­(diallyldimethylammonium) bis­(trifluoromethylsulfonyl)­imide
poly­([VBPy]­[NTf_2_]): poly­(1-vinyl-3-butylpyridinium bis­(trifluoromethylsulfonyl)­imide)
poly­([VCMim]­[Br]): poly­(1-carboxymethyl-3-vinylimidazolium bromide)
poly­([VCmim-COOH-*co*-AAm]­[Br]): 1-carboxymethyl-3-vinylimidazolium bromide-*co*-acrylamide
poly­([VCNMim]­[Br]): poly­(1-cyanomethyl-3-vinylimidazolium bromide)
poly­([VCNmim]­[Br]): poly­(1-cyanomethyl-3-vinylimidazolium bromide)
poly­([VCNmim-*co*-AAm]­[Br]): 1-cyanomethyl-3-vinylimidazolium bromide-*co*-acrylamide
poly­(4-aminoethyl-1- vinylimidazolium): bromide copolymerized with polyacrylamide
poly­(S-IL): poly­(styrene-based IL)
poly­(VAEmim-*co*-AAm]­[Br]): poly­(4-aminoethyl-1-vinylimidazolium)-*co*-polyacrylamide
poly­(VB-C4Py]­[NTf_2_]): poly­(1-vinyl-3-butylpyridinium bis­(trifluoromethylsulfonyl)­imide)
PP: polypropylene
PSF: polysulfone
PTMSP: poly­(1-trimethylsilyl-1-propyne)
PVA: poly­(vinyl alcohol)
PVAm: poly­(vinylamine)
PVDF: polyvinylidene fluoride
PVP: polyvinylpyrrolidone
PZEA-Sar: 2-(1-piperazinyl) ethylamine sarcosine
RAFT: reversible addition–fragmentation chain-transfer
SAPO-34: silicoaluminophosphate-34
SiC: silicon carbine
SILM: supported ionic liquid membrane system
SiNW: silicon nanowire
TFC: thin film composite
TOF: turnover frequency
WGS: water gas shift
wSBC: waste plastic styrene–butadiene copolymer
ZIF: zeolitic imidazolate frameworks
Zn­[NTf_2_]_2_: zinc bis­(trifluoromethanesulfonyl)­imide.
(P­([VBMI]­[Gly])): poly­[1-(p-vinylbenzyl)-3-methylimidazolium glycinate]
