# Supplementary material for: Progress, Challenges, and Opportunities in Ionic Liquid–Modified Polymer Membranes for CO2 Separation
Source: ACS Omega. 2026 Feb 19;11(8):12886–905. doi: 10.1021/acsomega.5c08808 (PMC12961509; doi:10.1021/acsomega.5c08808)
Supplement: Supplementary file 1 [file ao5c08808_si_001.pdf]

Progress, Challenges, and Opportunities in Ionic Liquid–Modified Polymer Membranes for CO_2 Separation

Julia A. Piotrowska†, Michael Harasek‡, and Katharina Bica-Schröder†*

†TU Wien, Institute of Applied Synthetic Chemistry, Getreidemarkt 9/163, 1060 Vienna, Austria

‡ TU Wien, Institute of Chemical, Environmental and Bioscience Engineering, Getreidemarkt 9/E166, 1060 Vienna, Austria

Supporting Information

Figure S1- Structures of the ionic liquids given in Table 1.

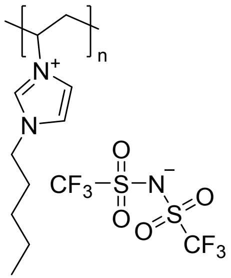

poly(1-vinyl-3-butylimidazolium bis(trifluoromethylsulfonyl)imide)

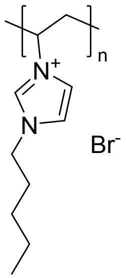

poly(1-vinyl-3-butylimidazolium bromide)

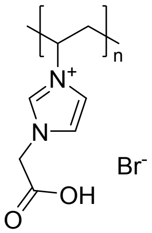

poly(1-carboxymethyl-3-vinylimidazolium bromide)

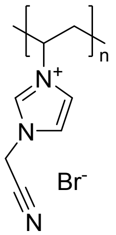

poly(1-cyanomethyl-3-vinylimidazolium bromide)

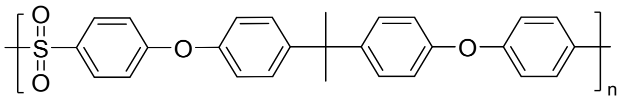

polysulfone

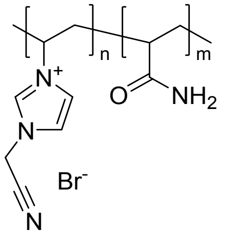

poly(1-cyanomethyl-3-vinylimidazolium bromide-co-acrylamide)

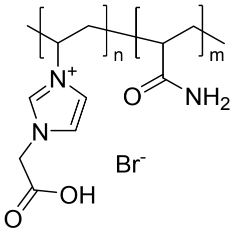

poly(1-carboxymethyl-3-vinylimidazolium bromide-co-acrylamide)

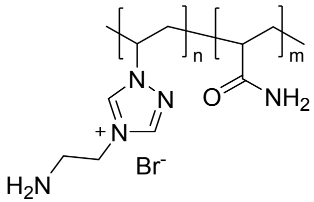

poly(4- aminoethyl-1-vinyl-1,2,4-triazolium) bromide-co-polyacrylamide
